# Liquid Bismuth Catalyst Enables High‐CO‐Selectivity in CO_2_ Hydrogenation

**DOI:** 10.1002/advs.202521489

**Published:** 2026-03-10

**Authors:** Xinxin Lu, Zun Guan, Xinyi Fu, Bin Chen, Zhongliang Huang, Riguang Zhang, Guowu Zhan

**Affiliations:** ^1^ Academy of Advanced Carbon Conversion Technology College of Chemical Engineering Huaqiao University Xiamen Fujian P. R. China; ^2^ Department of Biology Institute of Advanced Materials Hong Kong Baptist University Kowloon Tong Hong Kong SAR P. R. China; ^3^ State Key Laboratory of Clean and Efficient Coal Utilization College of Chemistry and Chemical Engineering Taiyuan University of Technology Taiyuan Shanxi P. R. China; ^4^ College of New Energy and Materials Engineering Shanxi Electronic Science and Technology Institute Linfen Shanxi P. R. China; ^5^ State Key Laboratory for Physical Chemistry of Solid Surfaces College of Chemistry and Chemical Engineering Xiamen University Xiamen Fujian P. R. China

**Keywords:** CO selectivity, CO_2_ hydrogenation, liquid bismuth catalysts, nanodroplet confinement, redox cycle

## Abstract

Here, we introduce a dynamic liquid‐bismuth (Bi) catalyst that exploits a reversible Bi^3+^/Bi^0^ redox cycle to address the challenge of poor CO selectivity in the reverse water‐gas shift (RWGS) reaction at moderate temperatures. Molten Bi nanodroplets were stabilized via in situ confinement during H_2_‐induced reduction of a BiVO_4_ precursor, resulting in their firm anchoring on a defective vanadium oxide (VO_x_) support while preserving dynamic surface mobility at 400°C, striking a balance between stability and fluidity. Combined mechanistic studies and DFT calculations revealed that Ni‐Bi dual sites on oxygen‐deficient VO_x_ facilitate an H_2_‐assisted redox mechanism: Bi sites enable direct CO_2_ dissociation and weak CO^*^ binding, favoring rapid CO desorption, while the Ni cocatalyst promotes H_2_ dissociation and induces electron enrichment of VO_x_ for CO_2_ adsorption. This work demonstrates the potential of liquid‐Bi‐based catalysts for selective and efficient CO_2_‐to‐syngas conversion.

## Graphical abstract

This work introduces a dynamic liquid bismuth catalyst for efficient CO_2_‐to‐syngas conversion. Stabilized molten Bi nanodroplets on a defective VO_x_ support enable a reversible Bi^3+^/Bi^0^ redox cycle at moderate temperatures. Mechanistic insights reveal that Ni‐Bi dual sites synergistically activate H_2_ and cleave CO_2_, leading to high CO selectivity.

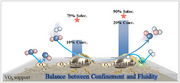

## 1 Introduction

The catalytic hydrogenation of CO_2_ via the reverse water‐gas shift (RWGS) reaction represents a pivotal route for CO_2_ utilization, producing syngas (CO/H_2_) as a feedstock for Fischer–Tropsch and methanol synthesis processes [1, 2]. As an endothermic process, the RWGS reaction is thermodynamically favored at elevated temperatures (500–700°C). However, operating at lower temperatures is desirable for improved energy efficiency. A key challenge arises from the competing, highly exothermic CO_2_ methanation reaction, which dominates below approximately 500°C and severely compromises CO selectivity [3, 4, 5]. Therefore, the development of catalysts that kinetically promote CO formation while suppressing over‐hydrogenation to methane is essential for efficient and selective CO_2_‐to‐syngas conversion.

Two distinct mechanisms govern RWGS catalysis: the formate pathway and the redox pathway [6, 7, 8, 9]. In contrast to the formate route with multi‐step proton‐coupled electron transfer, the redox pathway facilitates direct C═O bond cleavage, enabling significantly superior CO selectivity. Conventional transition metals (e.g., Cu, Ni, and Pt) typically exhibit strong CO binding, encountering inherent challenges in producing unwanted methane [10, 11, 12, 13]. While strategies such as surface encapsulation or alloying can moderate CO adsorption, they often result in low activity [14]. Bismuth‐based catalysts have recently emerged as promising redox mediators due to their innate resistance to over‐hydrogenation and favorable CO_2_ activation capability [15]. Metallic Bi participates in reversible redox cycles (Bi^3+^/Bi^0^), enabling CO_2_ reduction with high CO selectivity, as demonstrated in olefin synthesis and related CO_2_ hydrogenation processes [15]. However, the low melting point of Bi (275°C) poses a severe constraint: under typical catalytic temperatures (> 300°C), Bi sinters and aggregates, leading to rapid deactivation [16, 17].

Although supported liquid metal systems have been proposed to exploit the dynamic surface of low‐melting‐point metals, achieving a stable and finely dispersed liquid Bi phase remains particularly challenging. This is largely due to Bi's high surface tension and poor wettability on commonly used oxide supports, which often lead to droplet aggregation and catalyst deactivation [18, 19]. Our group recently developed a promising strategy to anchor active metal phases via the exsolution from a solid precursor, which generates nanostructured catalysts with robust metal‐support interactions and enhanced stability [20, 21, 22, 23]. Accordingly, bismuth vanadate (BiVO_4_) has attracted attention as a potential precursor, as its reduction under H_2_ can produce metallic Bi dispersed on a reducible VO_x_ support possessing abundant oxygen vacancies [24]. The multivalent nature of vanadium oxide facilitates vacancy formation, which is critical for CO_2_ activation. Nonetheless, the dynamic behavior of the Bi/VO_x_ interface under RWGS conditions, as well as the role of secondary metals in enhancing H_2_ activation without compromising CO selectivity, has not been fully elucidated.

In this work, we designed a dynamic liquid‐Bi catalyst stabilized on a defective VO_x_ support through H_2_‐induced exsolution from a BiVO_4_ precursor. Although exhibiting high surface mobility due to the low melting point of Bi, the resulting molten Bi nanodroplets remain confined and anchored to the VO_x_ surface. We further introduce trace Ni (1.6 wt.%) as a H_2_ dissociation promoter into the catalyst surface, creating a ternary Ni‐Bi/VO_x_ catalyst that significantly enhances RWGS performance. Through in situ diffuse reflectance infrared Fourier transform spectroscopy (DRIFTS) and density functional theory (DFT) simulations, we elucidate the H_2_‐assisted redox cycle involving Ni‐Bi dual sites on defective VO_x_: Bi enables direct CO_2_ dissociation and allows for weak CO^*^ adsorption, while the Ni cocatalyst facilitates H_2_ dissociation and induces electron‐rich VO_x_ for CO_2_ enrichment of the catalyst surface. This study demonstrates the potential of liquid‐Bi‐based catalysts for selective CO_2_ conversion to syngas, providing a new strategy for designing dynamic catalytic interfaces for renewable fuel and chemical production.

## 2 Results and Discussion

### 2.1 Fabrication and Structural Characterization of Bi/VO_x_ Catalyst

**FIGURE 1:**
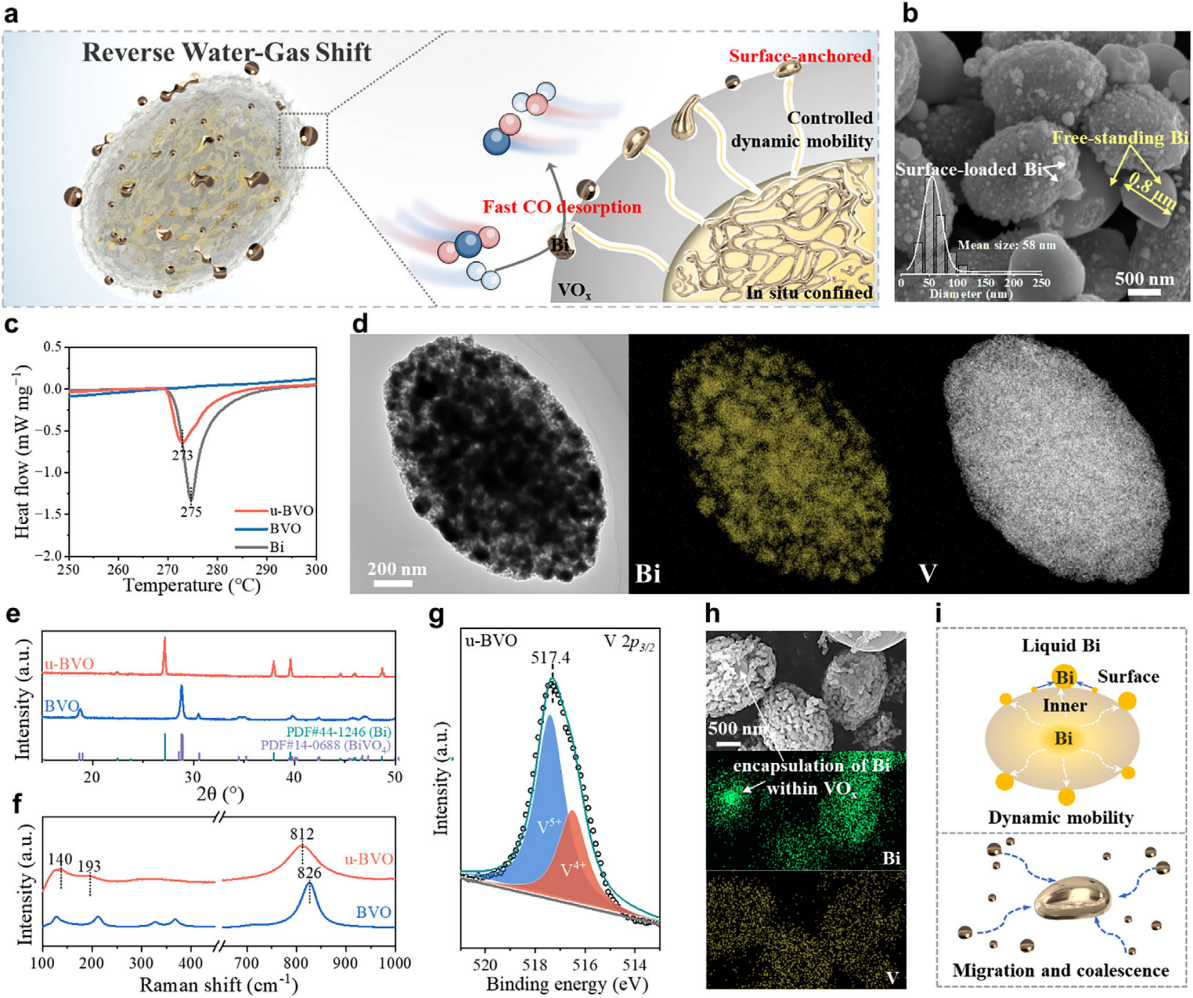
Microscopic characterization of u‐BVO catalyst. (a) illustration of the structure of the designed Bi/VO_x_ catalyst. (b) SEM image of u‐BVO (Inset: size statistic of Bi nanoparticle on the catalyst surface). (c) DSC analysis of Bi, BVO, and u‐BVO. (d) TEM image with corresponding elemental mappings of u‐BVO. (e) XRD patterns, and (f) Raman spectra of BVO and u‐BVO. (g) XPS profile of V 2*p* for u‐BVO. (h) SEM image corresponding to the elemental maps of BVO‐R600. (i) Illustration of liquid Bi nanodroplets migration and coalescence upon cooling.

The synthesis of Bi/VO_x_ catalysts via our “*solid precursor*” approach is schematically illustrated in Figure S1. Specifically, bismuth vanadate (BVO) precursors were first synthesized hydrothermally using polyvinylpyrrolidone (PVP) as a morphological template. These BVO precursors functioned as sacrificial templates, transforming under reducing reaction atmospheres to generate supported Bi/VO_x_ catalysts (denoted u‐BVO) via in situ metal exsolution (Figure 1a). Scanning electron microscopy (SEM) reveals that both pristine BVO and u‐BVO retain characteristic rice‐like morphologies (Figure S2a,b). Critically, u‐BVO surfaces display numerous particles confirmed as metallic bismuth by Energy‐dispersive X‐ray spectroscopy (EDS) mapping (Figure S2c).

Two distinct bismuth populations are identified (Figure 1b): surface‐anchored nanoparticles (ca. 58 nm, white arrow) and larger free‐standing particles (> 0.5 µm, yellow arrow). Differential scanning calorimetry (DSC, Figure 1c) reveals Bi's intrinsic melting point (∼275°C), confirming its molten state during catalytic operation (> 300°C). Consequently, the coexistence of these Bi morphologies arises from inherent molten‐Bi migration and coalescence upon cooling (room‐temperature). Importantly, despite macroscopic segregation, nano‐sized Bi particles remain tenaciously anchored, indicative of strong interfacial interactions with the VO_x_ support. This heterogeneous distribution is further resolved by Transmission electron microscopy (TEM) with correlated EDS elemental mapping (Figure 1d). High‐resolution TEM (HRTEM) imaging (Figure S2d) reveals lattice fringes at 3.28 Å, indexed to the Bi (012) plane. Additional spacings at 3.61 and 3.17 Å correspond to the (102) and (012) planes of Bi_2_O_3_, indicating partial surface oxidation of Bi domains. Notably, crystalline vanadium oxide phases remain undetected, consistent with an amorphous VO_x_ support structure as reported previously [24]. Based on ex situ SEM and TEM observations (Figure 1b,d), the liquid Bi catalyst solidifies into discrete particles distributed both on the surface and within the internal regions upon cooling; during the active reaction states, however, these particles would melt into liquid metals anchored by the VO_x_ support. It should be noted that a dynamic phase transition remains unobservable due to the absence of in situ high‐temperature SEM capabilities in this study.

X‐ray diffraction (XRD) analysis (Figure 1e) confirms the reduction‐induced transformation of BiVO_4_ precursors into metallic Bi (JCPDS No. 44–1246) under H_2_, while the VO_x_ support retains an amorphous structure. Raman spectroscopy (Figure 1f) corroborates the presence of VO_x_, revealing the characteristic V─O stretching vibration at 812 cm^−1^. Weak bands at 140 and 193 cm^−1^ suggest partial oxidation of VO_x_ to V_2_O_5_ [25]. Fourier‐transform infrared (FTIR) spectroscopy (Figure S3a) further supports this assignment, displaying characteristic V─O─V (748 and 515 cm^−1^) and V═O (986 cm^−1^) stretching vibrations associated with V_2_O_5_ formation [26, 27]. X‐ray photoelectron spectroscopy (XPS) probed surface chemical states, revealing mixed vanadium valences. Deconvolution of the V 2*p_3/2_* core level (Figure 1g) demonstrates the coexistence of substantial V^5+^ (517.4 eV) and V^4+^ (516.5 eV) species [28]. Additionally, the Bi 4*f* spectrum (Figure S3b) can be deconvolved into signatures for Bi^0^ (157.5 and 162.8 eV) and Bi^3+^ (159.4 and 164.7 eV). The high Bi^3+^/Bi^0^ intensity ratio (ca. 16:1) indicates significant surface oxidation of metallic Bi to Bi_2_O_3_ upon ambient exposure. Collectively, these results unequivocally demonstrate the in situ formation of liquid Bi nanodroplets dispersed on amorphous VO_x_ matrices derived from the controlled reduction of the BVO precursor under reduction conditions.

To elucidate the interfacial interactions between Bi and the VO_x_ support, BVO precursors were subjected to thermal treatment under an H_2_ atmosphere at 400°C and 600°C for 3 h, yielding catalysts denoted as BVO‐R400 and BVO‐R600, respectively. SEM and correlated EDS analyses (Figures S4 and S5) reveal complete decomposition of the BVO precursor at 600°C, generating abundant free‐standing Bi particles alongside fragmented VO_x_ domains. Crucially, partially preserved VO_x_ adopts a distinctive coral‐like morphology with extensive void structures (Figure S4c). EDS elemental mapping further identifies pronounced Bi enrichment within the core of these coral‐like VO_x_ aggregates (Figure 1h), confirming effective encapsulation of Bi domains. It should be clarified that Bi, in situ derived from BVO, is restricted to the pore network of the VO_x_ matrices, rather than being limited solely to the core. These observations demonstrate that the VO_x_ matrices spatially confine molten Bi, thereby stabilizing surface‐anchored Bi nanodroplets against coalescence and migration during high‐temperature reactions.

Accordingly, we propose a structural evolution pathway for Bi/VO_x_ formation from the BVO precursor (Figure S6), based on time‐resolved SEM observations (Figure S7) and the above discussion. During H_2_ reduction at 400°C, metallic Bi progressively exsolves from the BiVO_4_ lattice, nucleating as well‐dispersed nanodroplets anchored across the VO_x_ surface. These nanodroplets, composed of low‐melting‐point Bi (∼275°C), adopt a molten state under operational conditions (> 300°C). Molten Bi nanodroplets migrate and coalesce on both internal and external VO_x_ surfaces via a particle migration‐coalescence (PMC) mechanism (Figure 1i) [29]. Crucially, at catalytic temperatures (e.g., 400°C), liquid Bi nanodroplets remain anchored to VO_x_ yet exhibit dynamic inner and surface mobility, reflecting a balance between confinement and fluidity.

Inspired by the spatial confinement of molten Bi by the VO_x_ matrices, we hypothesized that precursor morphology modulates interfacial stability. We then synthesized three distinct BVO morphologies: rice‐like particles (BVO‐r, ∼1.9 µm long; Figure S2a), disc‐shaped crystals (BVO‐c, ∼0.7 µm diameter; Figure S8a,c), and sheet‐assembled aggregates (BVO‐a, diameter > 3 µm; Figure S8b,d). Despite morphological differences, all precursors exhibit nearly identical reduction profiles (Figures S9 and S10), confirming that structural evolution is governed by intrinsic chemistry rather than macroscopic form. These morphologically tailored precursors were subsequently used to interrogate structure‐function relationships in CO_2_ hydrogenation.

### 2.2 Fabrication of Ternary Ni‐Bi/VO_x_ Catalyst

A critical challenge lies in the oxygen incorporation during the redox cycle in the RWGS reaction, which can lead to the formation of surface oxides that degrade the catalyst's structural integrity or cause passivation [30]. To ensure sustained catalytic activity, the kinetics of oxide reduction must dominate over oxidation, a requirement that necessitates a high concentration of surface‐reactive hydrogen species. This can be effectively engineered by creating hydrogen spillover from specific dissociation sites to adjacent active surfaces, thus maintaining the redox cycle and stabilizing catalytic performance [31].

To incorporate active sites for H_2_ dissociation, the Ni cocatalyst was anchored onto bismuth vanadate (BVO) precursors via impregnation, followed by in situ reduction to yield the ternary Ni‐Bi/VO_x_ (denoted u‐Ni/BVO). Inductively coupled plasma‐optical emission spectrometer (ICP‐OES) quantified the elemental composition, revealing a Ni loading of 1.6 wt.%, alongside principal components of Bi (73.4 wt.%) and V (16.7 wt.%) (Table S1). XRD analysis of u‐Ni/BVO (Figure S11a) exhibited two weak diffraction peaks at 2θ = 43.3° and 44.4°, corresponding to the (012) plane of NiO and the (111) plane of metallic Ni, respectively. High‐resolution TEM (HRTEM) images (Figure S11b) further identified lattice fringes with a spacing of 2.10 Å, indexed to the NiO(200) plane. XPS results (Figure S11c) confirmed the coexistence of metallic Ni^0^ (853.0 eV) and Ni^2+^ (855.7 eV) [32]. Moreover, EDS mappings (Figure S12) demonstrated a highly dispersed distribution of Ni species. Collectively, these findings indicate the successful loading of well‐dispersed Ni nanoparticles, which predominantly exist as metallic Ni under H_2_‐rich conditions, with the observed NiO phase attributable to surface passivation upon air exposure. Additional characterization via SEM, TEM, XRD, and Raman spectroscopy (Figures S11–S13) consistently confirmed that the introduction of Ni cocatalyst does not perturb the structural evolution of the Bi/VO_x_ support during reduction.

**FIGURE 2:**
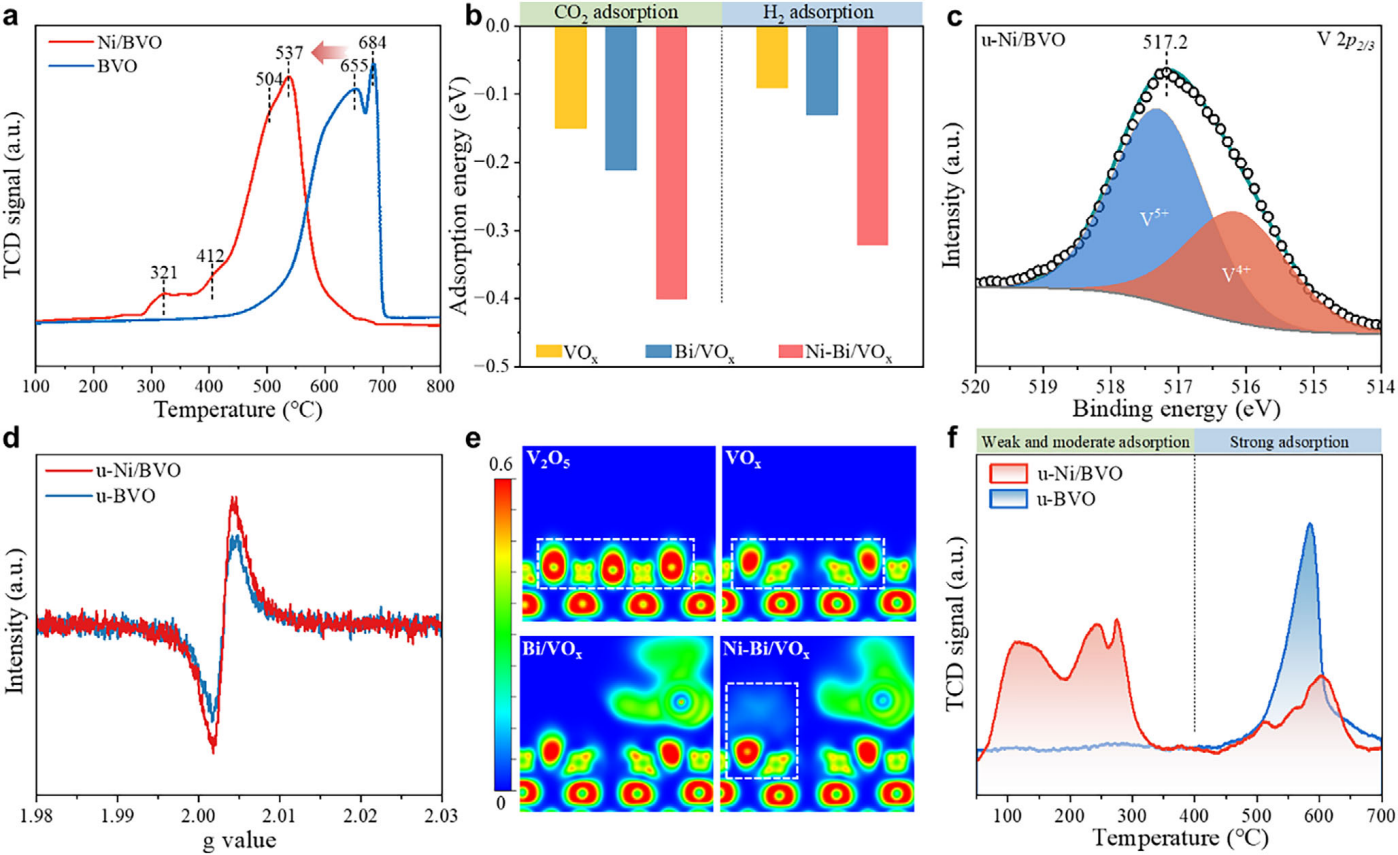
Characterization of u‐Ni/BVO catalysts. (a) H_2_‐TPR profiles of BVO and Ni/BVO precursors. (b) The adsorption free energies of CO_2_ and H_2_ over VO_x_, Bi/VO_x_, and Ni‐Bi/VO_x_. (c) XPS profile of V 2*p* for u‐Ni/BVO. (d) EPR spectra of u‐BVO and u‐Ni/BVO. (e) The electron localization function of V_2_O_5_, VO_x_, Bi/VO_x_, and Ni‐Bi/VO_x_. (f) CO_2_‐TPD profiles of u‐BVO and u‐Ni/BVO.

The role of Ni cocatalyst in modulating the reducibility of BVO was investigated by H_2_ temperature‐programmed reduction (H_2_‐TPR). As shown in Figure 2a, reduction peaks at 321°C (*α*‐NiO, weak metal‐support interaction) and 412°C (*β*‐NiO, moderate interaction) emerge [33, 34]. Critically, Ni incorporation lowers the primary reduction temperature of BiVO_4_ from 684°C to 537°C, consistent with H_2_ spillover from Ni to BVO [35]. This demonstrates that H_2_ dissociation at Ni sites generates active hydrogen species (H^*^), which subsequently migrate to the BVO surface, accelerating the reduction of Bi^3+^ (from BiVO_4_ precursor) to metallic Bi^0^. Furthermore, compared with u‐BVO, the enhanced HD signal on u‐Ni/BVO during the H‐D isotope exchange experiment provides direct evidence for the crucial role of Ni sites in H_2_ dissociation (Figure S14). Theoretically, H_2_ adsorption energy increases sequentially (Figure 2b): VO_x_ (‐0.09 eV) < Bi/VO_x_ (‐0.13 eV) < Ni‐Bi/VO_x_ (‐0.32 eV), with corresponding electron transfers of 0.003, 0.018, and 0.027 |*e*|, respectively (Figure S15). These results establish Ni sites as pivotal centers for H_2_ dissociation.

XPS analysis (Figure 2c) reveals a 0.2 eV decrease in the V 2*p_3/2_* binding energy for u‐Ni/BVO (517.2 eV) relative to u‐BVO (517.4 eV), signifying electron enrichment on the VO_x_ support induced by Ni incorporation. This electronic modulation is corroborated by Raman spectra, where the V─O stretching vibration shifts from 812 to 810 cm^−1^ (Figure S13) [36, 37]. Deconvolution of the O 1*s* spectrum (Figure S16) resolves three distinct states: lattice oxygen (O_L_), oxygen vacancies (O_V_), and surface hydroxyl groups (O_A_). Notably, u‐Ni/BVO exhibits a higher oxygen vacancy concentration (18.0%) than u‐BVO (15.0%), consistent with enhanced electron paramagnetic resonance (EPR) intensity at g = 2.001 (Figure 2d). Both O_L_ and O_V_ components in u‐Ni/BVO undergo subtle shifts toward lower binding energies (O_L_: 530.4 vs. 530.2 eV; O_V_: 532.2 vs. 532.1 eV), confirming electron transfer from Ni^0^ to VO_x_ via interfacial Ni‐O‐V linkages [38].

Electron localization function (ELF) mapping (Figure 2e; Figure S17) visualizes pronounced electron accumulation near interfacial O_V_ in Ni‐modified catalysts. This localized electron density enhances the electron‐donating capacity of VO_x_, a critical feature for CO_2_ adsorption. Bader charge analysis quantifies stronger charge transfer (0.041 |*e*|) from Ni‐Bi/VO_x_ catalyst to adsorbed CO_2_ versus isolated VO_x_ or Bi/VO_x_ (Figure S18). Consequently, CO_2_ adsorption free energy intensifies from ‐0.15 eV (VO_x_) and ‐0.21 eV (Bi/VO_x_) to ‐0.40 eV on Ni‐Bi/VO_x_, highlighting Ni‐promoted CO_2_ adsorption on VO_x_ (Figure 2b). Experimentally, CO_2_ temperature‐programmed desorption (CO_2_‐TPD) confirms enhanced CO_2_ adsorption capacity in Ni‐containing catalysts (Figure 2f). Thus, the Ni cocatalyst exhibits a bifunctional mechanism: it facilitates H_2_ dissociation while concurrently inducing an electron‐rich VO_x_ surface that promotes CO_2_ enrichment on the surface.

### 2.3 Catalytic Performance for CO_2_ Thermal Hydrogenation

**FIGURE 3:**
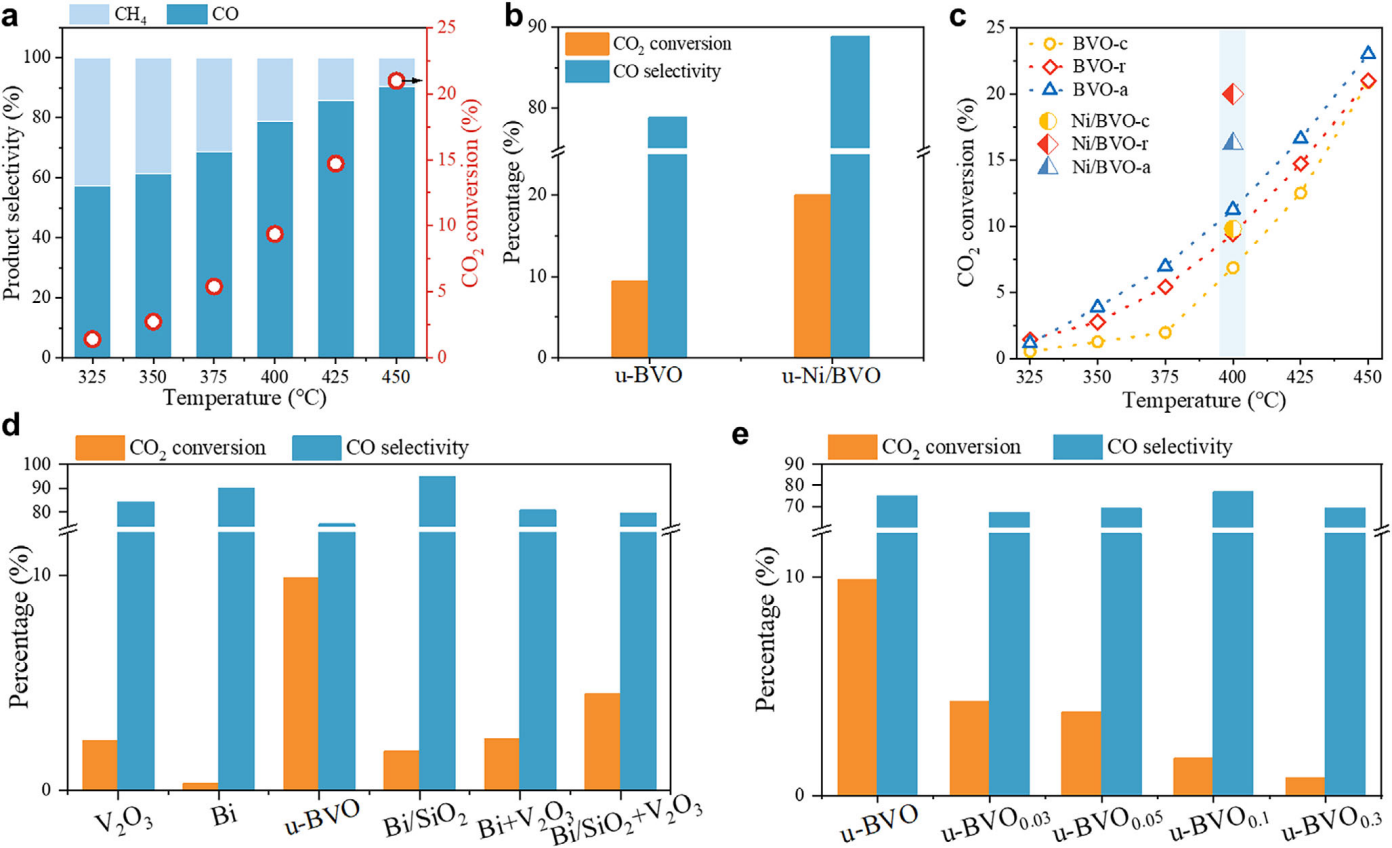
Catalytic performance for CO_2_ hydrogenation. (a) CO_2_ conversion and product selectivity of u‐BVO. (b) CO_2_ conversion and CO selectivity for u‐BVO and u‐Ni/BVO. (c) CO_2_ conversion for the as‐prepared catalysts with different precursor morphologies. (d,e) CO_2_ conversion and CO selectivity for (d) Bi‐based or V‐based catalysts, and (e) HCl post‐treated catalysts.

The CO_2_ thermal hydrogenation performance of u‐BVO was evaluated at 1 MPa and 8000 mL·g_cat_^−1^·h^−1^ over 325–450°C (Figure 3a). CO_2_ conversion increased with temperature, yielding CO predominantly alongside minor CH_4_. The u‐BVO catalyst achieved a CO_2_ conversion of 10.0% with a CO selectivity of 79.0% at 400°C. Upon incorporation of Ni at an optimal loading of 1.6 wt.%, the CO_2_ conversion increased to 20.0% while the CO selectivity rose to 90.0% (Figure 3b). This enhancement arises from the hydrogen‐rich environment generated by H_2_ dissociation on Ni sites, along with improved CO_2_ enrichment on the electron‐rich VO_x_ surface promoted by Ni. The fact that CO_2_ conversion showed limited dependence on Ni content, while CO selectivity remained consistently near 90.0% (Figure S19), suggests that the reaction likely follows a direct CO formation pathway. In contrast, a formate‐mediated mechanism would be expected to exhibit stronger sensitivity to H^*^ availability, which is modulated by Ni concentration during hydrogenation steps. Although the overall activity does not surpass that of leading Ni‐based catalysts (Table S2), these results constitute a significant demonstration of applying a liquid bismuth strategy to thermocatalytic CO_2_ hydrogenation while maintaining high CO selectivity.

Catalyst morphology modulated activity: sheet‐assembled BVO‐a achieved the highest CO_2_ conversion (11.0%) at 400°C, surpassing rice‐like BVO‐r (10.0%) and disc‐like BVO‐c (7.0%) (Figure 3c; Figure S20). Regardless of the catalyst morphology, the product distribution remained largely similar, yielding a CO selectivity of ∼78.0% and a CH_4_ selectivity of ∼22.0% at 400°C. Scherrer analysis (Table S3) confirms severe PMC of Bi upon cooling in u‐BVO‐c, yielding larger Bi particles (60.8 nm vs. 47.9 nm for u‐BVO‐r, 47.8 nm for u‐BVO‐a), while the coral‐like VO_x_ framework in BVO‐a/BVO‐r suppresses Bi sintering through spatial confinement. Ni immobilization further enhanced CO_2_ conversion (400°C): Ni/BVO‐c (9.8%) < Ni/BVO‐a (16.2%) < Ni/BVO‐r (20.0%) with comparable CO selectivity (∼90%). Negligible enhancement in Ni/BVO‐c arises from its stacked disc‐like structure, which fails to prevent the coalescence of molten Bi (Table S3). This highlights that ensuring sufficient and accessible Bi active sites is crucial for achieving CO_2_ conversion in this system, which can be modulated by support morphology.

Control experiments elucidate critical Bi‐VO_x_ synergy (Figure 3d). Catalysts lacking integrated Bi/VO_x_ interaction exhibited low activity (<5.0% CO_2_ conversion), with activity trending: Bi/SiO_2_+V_2_O_3_ (physical mixture)> Bi+V_2_O_3_ (physical mixture)> V_2_O_3_> Bi/SiO_2_> Bi. Negligible activity of Bi (fully reduced to metallic Bi at 400°C; Figures S21 and S22) and sintered Bi particles in BVO‐R600 (4.0% CO_2_ conversion) confirm that VO_x_‐anchored Bi nanodroplets, not free‐standing Bi, serve as primary active sites. Low activity of Bi dispersed on amorphous SiO_2_ from Bi_2_SiO_5_ (2.0% CO_2_ conversion; Figures S21 and S23) and measurable CO_2_ conversion on V_2_O_3_ (2.4% CO_2_ conversion; Figures S21 and S22) suggest that VO_x_ supports provide secondary active sites for CO_2_ reduction, though their contribution is subordinate to Bi‐VO_x_ synergy. These results unequivocally establish that molten Bi nanodroplets anchored on VO_x_ dominate CO_2_ reduction to CO, with Ni optimizing H^*^ flux and interfacial electron transfer.

To further elucidate the dual role of VO_x_ in stabilizing Bi nanodroplets and activating CO_2_, we subjected u‐BVO to selective surface Bi etching using HCl solutions (concentration: 0.03–0.3 m), yielding catalysts denoted u‐BVO_n_ (*n* = HCl concentration; Figures S24 and S25). Increasing HCl concentrations from 0.03 to 0.3 m progressively diminished CO_2_ conversion from 4.3% to 0.8% (400°C, 1 MPa), while CO selectivity remained stable (∼75%; Figure 3e). Comprehensive characterization (Raman, SEM, and digital photos in Figures S25–S27) reveals that HCl etching induces two parallel degradation pathways: (i) dissolution of the coral‐like VO_x_ framework and (ii) accelerated agglomeration of Bi into micron‐scale particles. Crucially, the extent of activity loss directly correlates with the depletion of surface‐anchored Bi nanodroplets, confirming their indispensability for CO_2_ activation. These results demonstrate that VO_x_ provides dual functionality: (i) spatially confining molten Bi to maintain a balance between confinement and fluidity and (ii) enriching CO_2_ on its surface to enhance the reaction probability of Bi and CO_2_.

Temperature‐swing cycling experiments demonstrated the regenerability of both u‐BVO and u‐Ni/BVO catalysts, attributable to Bi condensation/remelting dynamics (Figure S28). However, over repeated cycles, the optimized u‐Ni/BVO catalyst experienced a slight activity loss, with CO_2_ conversion decreasing from 20.0% to 17.0% by the third cycle. To further evaluate operational durability, long‐term stability tests were conducted under standard reaction conditions (Figure S29,). Both catalysts exhibited performance degradation over the 48 h test. Specifically, the u‐BVO catalyst showed a 16.0% activity loss, with CO_2_ conversion eventually stabilizing near 8.0%. In contrast, u‐Ni/BVO underwent a more gradual activity reduction of 36.0%, yet its final conversion (12.5%) remained higher than that of u‐BVO, while maintaining CO selectivity around 90.0%. Although the Bi/VO_x_ phase composition remained unchanged in both catalysts (Figure S30), post‐reaction characterization revealed severe Bi aggregation in u‐Ni/BVO(48 h), as indicated by an increase in the average Bi particle size from 47.0 to 58.2 nm (calculated from XRD; Table S3). In comparison, u‐BVO showed no significant Bi aggregation (45.2 nm vs. 47.9 nm). These findings suggest that catalyst deactivation is primarily driven by the inherent aggregation of Bi. The presence of Ni exacerbates this process, likely by creating an H‐rich surface environment that enhances Bi atom mobility. It has been reported that reductive conditions can weaken metal‐support interactions and promote metal atom mobility, leading to catalyst deactivation [39]. Nonetheless, the relatively stable performance of u‐BVO underscores the importance of balancing Bi confinement and fluidity to sustain catalytic activity.

### 2.4 Mechanistic Insights Into CO_2_ Hydrogenation

**FIGURE 4:**
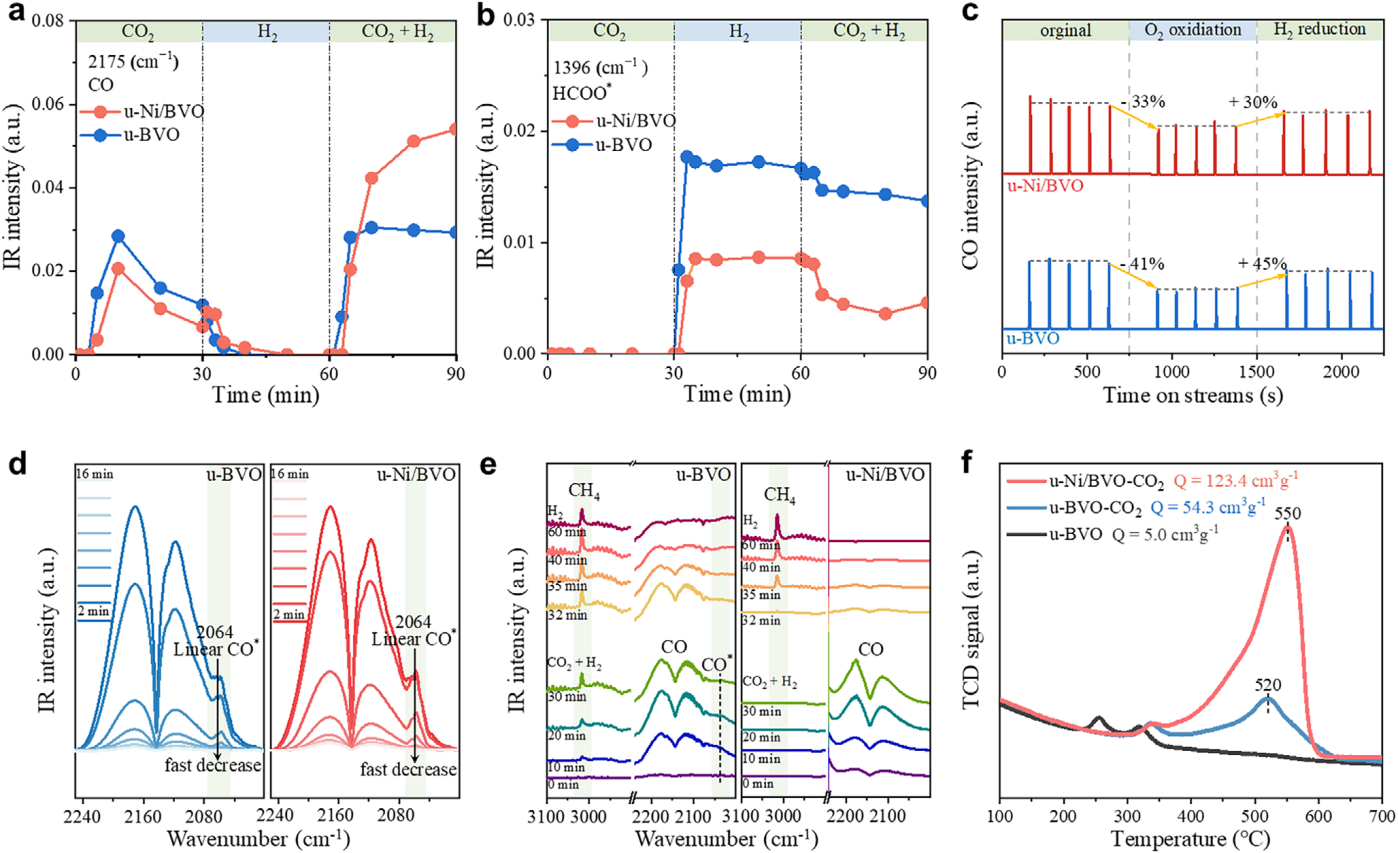
Revealing reaction mechanisms of u‐BVO and u‐Ni/BVO catalysts. (a,b) changes in the intensities of IR bands of (a) CO at 2175 cm^−1^, (b) formate species at 1396 cm^−1^. (c) CO intensity measured during CO_2_ pulse experiments at 400°C. (d) In situ DRIFT spectra of CO desorption at 40°C for u‐BVO and u‐Ni/BVO. (e) in situ DRIFT spectra at 400°C and 1 MPa for u‐BVO and u‐Ni/BVO. (f) H_2_‐TPR profiles of u‐BVO, u‐BVO‐CO_2_, and u‐Ni/BVO‐CO_2_.

In situ DRIFTS was then employed to probe the reaction intermediates over u‐BVO and u‐Ni/BVO catalysts (Figure S31). Catalysts were pre‐reduced in H_2_ (400°C, 3 h) and purged with N_2_ to clean surfaces. When CO_2_ gas was introduced, gaseous CO bands emerged at 2175 and 2115 cm^−1^ with comparable intensity for both catalysts (Figure 4a), owing to direct CO_2_ dissociation. Upon switching the feed from CO_2_ to H_2_, a distinct peak at 1396 cm^−1^, assigned to bidentate formate intermediate (HCOO^*^), appeared (Figure 4b) [40, 41, 42]. Critically, no decomposition of HCOO^*^ to CO was observed, ruling out the associative pathway [43, 44, 45, 46]. This confirms CO production predominantly via a redox mechanism, where CO_2_ dissociates directly on Bi sites, and H_2_ regenerates reduced states of the active site.

CO_2_ pulse experiments (Figure 4c) further validate this mechanism: CO_2_ pulses immediately generated CO, but subsequent catalyst oxidation suppressed CO yield. Activity was restored after H_2_‐mediated re‐reduction, demonstrating that H_2_ acts as a reductant for catalyst regeneration, not a direct HCOO^*^ hydrogenation agent. The intensified CO signal on u‐Ni/BVO (Figure 4a) thus arises from accelerated Bi/VO_x_ regeneration via H^*^ spillover from Ni sites, consistent with lower reaction onset temperatures in CO_2_/H_2_ temperature‐programmed surface reaction (TPSR experiment; Figure S32). Meanwhile, the linear CO^*^ adsorption at 2064 cm^−1^ vanished rapidly under N_2_ purge (Figure 4d), reflecting weak CO adsorption on u‐BVO and u‐Ni/BVO. This aligned with high CO selectivity, and Ni incorporation did not alter CO adsorption strength. As reported, weak CO adsorption on Bi sites is due to the intrinsic electronic properties of metallic Bi, which lacks a sufficient population of energetically suitable electrons for effective back‐donation into the 2π^*^ anti‐bonding orbital of CO [47].

High‐pressure in situ DRIFTS (Figure S33) identified additional reaction intermediates. In the case of the u‐BVO catalyst, a linear CO^*^ peak at 2031 cm^−1^ (transient intermediate) diminished over time, while HCOO^*^ (1396 cm^−1^) and CH_4_ (3015 cm^−1^) intensities increased (Figure 4e), indicating CO^*^ hydrogenation as the primary CH_4_ formation pathway. However, for u‐Ni/BVO, CH_4_ was detected only upon switching from CO_2_/H_2_ to pure H_2_ (Figure 4e). This demonstrates that H^*^‐mediated rapid regeneration of Bi/VO_x_ suppresses CO^*^ accumulation and over‐hydrogenation, favoring CO desorption.

Furthermore, H_2_‐TPR quantified the redox cycle dynamics of u‐BVO and u‐Ni/BVO catalysts (Figure 4f). Pre‐treated catalysts (u‐BVO‐CO_2_ and u‐Ni/BVO‐CO_2_) were exposed to a CO_2_ atmosphere after H_2_ reduction. Negligible H_2_ consumption (5 cm^3^·g^−1^) occurred on non‐oxidized u‐BVO, while oxidized u‐BVO‐CO_2_ consumed 49.3 cm^3^·g^−1^ H_2_ (peak at 520°C). These observations strongly support a catalytic redox cycle involving Bi droplets as the primary active sites (i.e., redox cycle, CO_2_ + Bi^0^ → CO + Bi_2_O_3_, H_2_ + Bi_2_O_3_ → H_2_O + Bi^0^). In the case of u‐Ni/BVO‐CO_2_, H_2_ consumption surged to 123.4 cm^3^·g^−1^ with a delayed reduction peak (550°C). This arises from a CO_2_‐enriched microenvironment around Bi^0^ sites created by Ni‐induced electron‐rich VO_x_. Additionally, the Bi_2_O_3_ overlayer formed during oxidation further stabilizes molten Bi droplets against coalescence [48].

### 2.5 DFT Calculations

**FIGURE 5:**
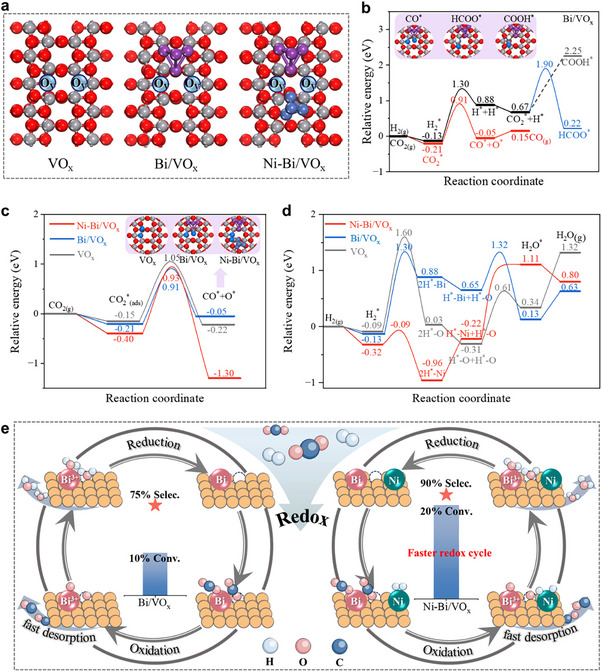
DFT calculations. (a) Three catalyst structures modeled VO_x_, Bi/VO_x_, and Ni‐Bi/VO_x_. (b–d) relative energy profiles of different pathways for (b) CO_2_ dissociation into CO^*^, HCOO^*^, and COOH^*^ at O_V_ site on Bi/VO_x_, (c) direct CO_2_ dissociation, (d) H_2_ dissociation and regeneration of O_V_ on VO_x_, Bi/VO_x_, and Ni‐Bi/VO_x_. (e) proposed RWGS reaction mechanism over Bi/VO_x_ and Ni‐Bi/VO_x_ catalysts (H_2_‐assisted redox cycle).

DFT calculations were conducted to unravel the role of O_V_ in the H_2_‐assisted redox mechanism. To emulate the experimental catalyst structures, oxygen‐deficient V_2_O_5_ (001) surfaces with controlled O_V_ concentrations were modeled as VO_x_, Bi/VO_x_, and Ni‐Bi/VO_x_ systems (Figure 5a; Figure S34). In these models, a static Bi_4_ cluster was employed as a targeted computational probe to capture the electronic structure of low‐coordination surface atoms, which represent the most active sites on liquid Bi nanodroplets rather than simulating the entire dynamic droplet. This simplification is justified by the intrinsic atomic‐scale layering and short‐range order characteristic of liquid metal surfaces, which confer transient local rigidity and enable meaningful electronic structure analysis using static cluster models [49, 50, 51]. The formation energy of an additional O_V_ was calculated to evaluate the ease of O_V_ generation, following the trend: Bi/VO_x_ (2.89 eV) < Ni‐Bi/VO_x_ (3.16 eV) < VO_x_ (3.24 eV). These results indicate that Bi nanoparticles promote oxygen lattice activation and facilitate O_V_ formation on the VO_x_ support. The presence of Ni further fine‐tunes the oxygen vacancy formation energy, reflecting its role in modulating the redox properties of the catalytic interface. This computational approach provides atomic‐level insight into the synergistic roles of Bi and Ni in enhancing the redox activity and stability of the VO_x_‐supported catalyst.

Reaction free energy profiles for CO_2_ reduction pathways are shown in Figure 5b and Figure S35 and S36. The associative pathway (CO_2_^*^ + H^*^ → HCOO^*^) on Bi/VO_x_ requires a high H_2_ dissociation barrier (1.43 eV) and a kinetic barrier of 1.23 eV. In contrast, direct CO_2_ dissociation at O_V_ sites (CO_2_^*^ → CO^*^ + O^*^) exhibits a lower kinetic barrier (1.12 eV), thermodynamically and kinetically favoring this route over HCOO^*^ or COOH^*^‐mediated paths, consistent with the above‐mentioned in situ DRIFTS data. The dissociated CO^*^ adsorbs on adjacent Bi sites, but desorbs rapidly due to weakly adsorbed CO on adjacent Bi sites (*E*_ads_ = −0.20 eV), aligning with high experimental CO selectivity. Incorporation of Bi or Ni‐Bi further reduces the direct CO_2_ dissociation barrier at O_V_ from 1.05 eV (VO_x_) to 0.91 eV (Bi/VO_x_) and 0.93 eV (Ni‐Bi/VO_x_) (Figure 5c; Figure S37). Although CO binds strongly on Ni sites (*E*_ads_ = −1.60 eV), abundant neighboring Bi sites attenuate adsorption strength, ensuring efficient CO desorption after its production.

H_2_ dissociation barrier decreases dramatically at Ni sites (0.23 eV) versus Bi (1.43 eV) and VO_x_ (1.69 eV) (Figure 5d; Figure S38), confirming Ni as the dominant H_2_ activation center. Active H^*^ species migrate from Ni to the Bi/VO_x_ surface, participating in redox cycles. The regeneration of O_V_ via H_2_O^*^ formation proceeds most efficiently at Bi─O sites (barrier: 0.67 eV) compared to Ni─O (1.33 eV) and O─O (0.92 eV). Therefore, the synergy of Ni‐Bi facilitates the O_V_ regeneration on VO_x_.

Based on the analysis, we propose a comprehensive H_2_‐assisted redox mechanism for the RWGS reaction over the ternary Ni‐Bi/VO_x_ catalyst, as illustrated in Figure 5e. The mechanism involves two primary steps: (a) CO_2_ enrichment and dissociation, where CO_2_ is concentrated on the Ni‐induced electron‐rich VO_x_ surface and undergoes direct dissociation at Bi and O_V_ sites, consuming metallic Bi and filling vacancies (CO_2_ + Bi^0^ → CO + Bi_2_O_3_; CO_2_ + O_V_ → CO + O^*^); and (b) reductive regeneration, where H_2_ dissociates at Ni sites to generate mobile H^*^ species that reduce the oxidized Bi and replenish the oxygen vacancies, producing H_2_O (H_2_ + Bi_2_O_3_ → H_2_O + Bi^0^; 2H^*^ + O^*^‐O_V_ → H_2_O^*^ + O_V_). Water desorption subsequently regenerates the oxygen vacancies, completing the catalytic cycle. In this scheme, the Ni cocatalyst plays a dual role: (i) it induces electron enrichment of the VO_x_ support to enhance CO_2_ adsorption, and (ii) it provides a continuous supply of H^*^ for the rapid regeneration of active Bi sites. Concurrently, Bi facilitates weak CO adsorption, enabling high CO selectivity and sustained activity. The high Bi content (73.4 wt.%) and the dense population of Bi nanodroplets on the VO_x_ surface favor a direct redox pathway. Furthermore, the Ni‐promoted electron‐rich VO_x_ support creates a CO_2_‐enriched microenvironment at the interface, enhancing the interaction between CO_2_ and the active Bi/O_V_ sites (Figure S39).

The molten state of the Bi nanodroplets serves as a central, dual‐function feature that governs both the high efficiency of CO_2_ reduction and the catalyst's eventual deactivation. On one hand, the inherent fluidity of the liquid Bi phase enables continuous surface renewal, promoting a highly dynamic Bi^3+^/Bi^0^ redox cycle essential for CO_2_ decomposition [18]. This behavior is also observed in other liquid metal systems such as In/In_2_O_3_ and Ga/Ga_2_O_3_ [19, 52]. On the other hand, the high surface energy and mobility of the liquid droplets drive particle migration and coalescence (PMC), leading to irreversible agglomeration and consequent loss of active surface area over time.

## 3 Conclusion

In summary, this work demonstrates a rational strategy for synthesizing molten Bi‐based catalysts that achieve effective thermal CO_2_ hydrogenation to CO. Liquid Bi nanodroplets, spontaneously formed and spatially confined on VO_x_ matrices, serve as robust active sites for CO_2_ activation. Multiscale characterization and controlled experiments reveal that metallic Bi dynamically exsolves from BiVO_4_ precursors during reaction, forming molten Bi nanodroplets dispersed on amorphous VO_x_ matrices by strong Bi/VO_x_ interactions. We establish an exclusive H_2_‐assisted redox mechanism for the RWGS reaction: (i) direct CO_2_ dissociation occurs at Bi sites and O_V_ on VO_x_; (ii) the incorporation of Ni cocatalyst accelerates Bi and O_V_ regeneration via H‐species spillover, while simultaneously enhancing CO_2_ enrichment on the surface of VO_x_. Critically, high CO selectivity stems from two synergistic effects: weak CO adsorption on metallic Bi^0^ sites, and efficient O_V_ regeneration mediated by Bi─O interfaces. This study serves as an initial investigation into the application of liquid metals in thermocatalytic CO_2_ hydrogenation. Although the catalytic performance, particularly in terms of CO_2_ conversion, does not yet rival that of state‐of‐the‐art RWGS catalysts operating at high space velocities, it highlights the importance of meticulously engineering the metal‐support interface for developing high‐performance liquid metal catalysts. The viability of supported liquid metal systems hinges on two critical factors: (a) achieving uniform dispersion of liquid metal droplets across the support and (b) ensuring their stability under reaction conditions. Therefore, addressing these interfacial challenges will be the primary focus of our ongoing efforts to optimize liquid Bi‐based catalysts for efficient RWGS conversion.

## Conflicts of Interest

The authors declare no conflict of interest.

## Supporting information




**Supporting File**: advs74745‐sup‐0001‐SuppMat.docx.

## Data Availability

The data that support the findings of this study are available from the corresponding author upon reasonable request.
