# Supplementary material for: Liquid Bismuth Catalyst Enables High‐CO‐Selectivity in CO2 Hydrogenation
Source: Adv Sci (Weinh). 2026 Mar 10;13(28):e21489. doi: 10.1002/advs.202521489 (PMC13185877; doi:10.1002/advs.202521489)
Supplement: Supplementary file 1 — Supporting File: advs74745‐sup‐0001‐SuppMat.docx. [file ADVS-13-e21489-s001.docx]

***Supporting Information***

Liquid Bismuth Catalyst Enables High-CO-Selectivity in CO_2_ Hydrogenation

Xinxin Lu^#^, Zun Guan^#^, Xinyi Fu, Bin Chen, Zhongliang Huang, Riguang Zhang*, and Guowu Zhan*

X. Lu^#^, X. Fu, B. Chen, G. Zhan
Academy of Advanced Carbon Conversion Technology, College of Chemical Engineering, Huaqiao University, 668 Jimei Avenue, Xiamen, Fujian, 361021, P. R. China
E-mail: gwzhan@hqu.edu.cn

Z. Guan^#^, R. Zhang
State Key Laboratory of Clean and Efficient Coal Utilization, College of Chemistry and Chemical Engineering, Taiyuan University of Technology, Taiyuan, Shanxi, 030024, P. R. China
E-mail: zhangriguang@tyut.edu.cn

Z. Guan^#^
College of New Energy and Materials Engineering, Shanxi Electronic Science and Technology Institute, Linfen, Shanxi, 041000, P. R. China

X. Lu^#^
Department of Biology, Institute of Advanced Materials, Hong Kong Baptist University, Kowloon Tong, Hong Kong SAR, 999077, P. R. China

Z. Huang
State Key Laboratory for Physical Chemistry of Solid Surfaces, College of Chemistry and Chemical Engineering, Xiamen University, Xiamen, Fujian, 361005, P. R. China

[^#^] These two authors contributed equally to this work.

## Chemicals

The following chemicals were used as received without further purification: ammonium metavanadate (NH_4_VO_3_, 99%, Alfa Aesar), bismuth nitrate pentahydrate (Bi(NO_3_)_3_·5H_2_O, 98%, Alfa Aesar), bismuth chloride (BiCl_3_, 99.7%, Aladdin), polyvinylpyrrolidone ((C_6_H_9_NO)_n_, 99.8%, Adamas-Beta), citric acid (C_6_H_8_O_7_, 99.5%, Titan), ammonium hydroxide (NH_4_OH, 25-28%, Sinopharm), ethylene diamine tetraacetic acid (EDTA, 99%, Adamas-Beta), nickel acetylacetonate (C_10_H_14_NiO_4_, 98%, Adamas-Beta), ethyl alcohol (C_2_H_6_O, 99.7%, Sinopharm), ethylene glycol (C_2_H_6_O_2_, 99.7%, Sinopharm), nitric acid (HNO_3_, 67%, Xilong), sodium hydroxide (NaOH, 98%, Titan), hydrochloric acid (HCl, 37%, Sinopharm), ethanolamine (C_2_H_7_NO, 99%, Aladdin), bismuth oxide (Bi_2_O_3_, 99.9%, Aladdin), vanadium oxide (V_2_O_5_, 98%, Sigma-Aldrich), sodium metasilicate nonahydrate (Na_2_SiO_3_·9H_2_O, AR grade, Sinopharm). The ultrapure water (UP, 18.5 MΩ cm) was obtained from a Kerton lab DAY-20 water polishing system.

**Synthesis of BiVO_4_ (BVO-x) precursors.** Three distinct BiVO_4_ precursors with varying morphologies and sizes were synthesized through modified hydrothermal procedures, as documented in previous studies.[1-4] **Synthesis of BVO-c**. Under adequate stirring, 0.5 mmol of BiCl_3_ was added to 50 mL of deionized water, followed by the addition of 0.5 mmol of NH_4_VO_3_. Before the hydrothermal reaction at 160 °C for 12 h, the aqueous ethanolamine solution (1 M) was added dropwise to the suspension with stirring until the pH value reached around 6.[1] **Synthesis of BVO-r**. 0.4 mmol of Bi(NO_3_)_3_·5H_2_O and 0.9 g of polyvinylpyrrolidone in 25 mL of ethylene glycol were prepared as solution A. Solution B was prepared by dissolving 0.4 mmol of NH_4_VO_3_ in 15 mL of deionized water at 70 °C. The as-prepared solutions A and B were mixed thoroughly, followed by hydrothermal treatment at 180 °C for 9 h.[4] **Synthesis of BVO-a**. Solution A was prepared by dissolving 4 mmol of Bi(NO_3_)_3_·5H_2_O and 4 mmol of citric acid in 15 mL of ethylene glycol. Simultaneously, solution B was prepared by dissolving 4 mmol of NH_4_VO_3_ in 25 mL of deionized water at 70 °C. The combined solutions were vigorously stirred for 1 h at room temperature, followed by the injection of 1 mL of NH_4_OH. Finally, the mixture was hydrothermally treated at 180 °C for 24 h.[3] **Synthesis of BVO-b**. 0.5 mmol of Bi(NO_3_)_3_·5H_2_O and 0.1 g of EDTA were dissolved in 15 mL of 1 M HNO_3_ under agitation. 0.5 mmol of NH_4_VO_3_ was dissolved in 15 mL of 1 M NaOH. Afterward, these as-prepared solutions were mixed uniformly for 30 min, followed by pH adjustment to approximately 7 using 0.5 M NaOH. The final mixture was transferred into a Teflon-lined autoclave and crystallized at 180 °C for 8 h.[2] The resulting BiVO_4_ precursors (BVO-c, BVO-r, BVO-a, BVO-b) were collected by centrifugation, washed sequentially with deionized water and ethanol (three times each), and dried at 60 °C overnight for subsequent characterization and use. Notably, the BVO mentioned in the text is BVO-r.

**Synthesis of Ni/BiVO_4_-x (Ni/BVO-x) precursors.** Briefly, 200 mg of uncalcined BVO-x was dispersed in 20 mL of Ni(acac)_2_ solution (17.5 mg Ni(acac)_2_ in 20 mL of ethanol) with sonication for 30 min. The mixture was stirred at 90 °C to evaporate the ethanol until the solid was collected. Subsequently, the collected sample was calcined at 500 °C for 2 h.

**Synthesis of** **Bi_2_SiO_5_ precursor.** Bi_2_SiO_5_ was synthesized using a modified method.[5] Solution A was prepared by dissolving 3 mmol of Bi(NO_3_)_3_·5H_2_O in 30 mL of ethylene glycol. Meanwhile, 1.5 mmol of Na_2_SiO_3_·9H_2_O was dissolved in 30 mL of deionized water as solution B. Next, solution A was mixed with solution B while being continuously stirred. The suspension was treated with a dropwise addition of 2 M NaOH solution until the pH value reached approximately 10, followed by hydrothermal treatment at 200 °C for 10 h. After crystallization, the as-synthesized samples were collected by centrifugation, washed, and dried at 60 °C overnight.

**Post-treatment of the used catalyst.** Following the reduction of the BVO-r precursors in H_2_ atmosphere for 3 h, 350 mg of the resultant samples underwent an HCl soaking treatment (concentration: 0.03 M, 0.05 M, 0.1 M, and 0.3 M, respectively) under stirring for 12 h at room temperature.

## Characterization methods

The crystalline structures of the samples were determined by recording XRD patterns with Cu Kα radiation (Rigaku SmartLab X-ray Diffractometer). The crystal size was estimated using the Scherrer equation. The morphologies and particle sizes were characterized with Scanning Electron Microscope (SEM, Hitachi SU5000) and Transmission Electron Microscopy (TEM and HRTEM, JEOL JEM 2100F). Additionally, corresponding Energy-Dispersive X-ray Spectrometry (EDS) was employed to determine the distribution of elements within the samples. The Ni content in all samples was quantified by Inductively Coupled Plasma Optical Emission Spectrometry (ICP-OES, Varian 720-ES). Raman (Renishaw) spectra were obtained using an inVia Reflex spectrometer equipped with a 532 nm laser. Fourier-Transform Infrared (FTIR, Thermo Nicolet iS50, US) spectra were recorded using a spectrometer. Chemical valence properties were analyzed using an X-ray Photoelectron Spectrometer (XPS, Thermo ESCALAB 250XI) with an Al Kα X-ray excitation source. XPS data were calibrated based on the binding energy of C 1*s* at 284.8 eV. The melting temperature (Tm) was determined by Differential Scanning Calorimetry (DSC, TA). Electron Paramagnetic Resonance spectra (EPR) were collected on a Bruker A300. Temperature-Programmed Reduction of Hydrogen (H_2_-TPR) test and Temperature-Programmed Desorption of Carbon Dioxide (CO_2_-TPD) were conducted using an automated chemisorption analyzer (Auto Chem II 2920). For the H_2_-TPR test, 30 mg of the synthesized sample was initially heated from room temperature to 300 °C (10 °C min^−1^) under a He flow (20 mL min^−1^) for 1 h. After cooling to 50 °C, a continuous flow of 10% H_2_/Ar (20 mL min^−1^) was flowed over the sample, while the temperature was increased from 50 to 800 °C at 10 °C min^−1^. For H_2_-TPR experiments over the CO_2_-treatment sample, 30 mg of the sample was pretreated under a flow of 10% H_2_/Ar (20 mL min^−1^) at 400 °C for 3 h. The gas was then switched to a pure CO_2_ flow (5 mL min^−1^) at 400 °C for another 3 h. He (20 mL min^−1^) was subsequently introduced to displace CO_2_ at 400 °C for 1 h. After cooling to 50 °C, H_2_ consumption was monitored while the temperature increased from 50 to 800 °C at 10 °C min^−1^. In the CO_2_-TPD experiments, 50 mg of sample was initially treated with a 10% H_2_/Ar flow (20 mL min^−1^) at 400 °C for 3 h to achieve full reduction. The gas was then switched to a He flow (20 mL min^−1^) for 1 h to remove impurities. After cooling to 50 °C, the sample was exposed to pure CO_2_ (20 mL min^−1^) for 1 h to achieve saturated adsorption. Subsequently, He flow (20 mL min^−1^) was introduced to remove physically adsorbed CO_2_ from the sample surfaces. Finally, the temperature was raised from 50 to 700 °C at 10 °C min^−1^ under a He flow (20 mL min^−1^).

## Catalytic performance evaluation method

The catalytic CO_2_ hydrogenation performance was investigated in a continuous flow fixed-bed quartz glass tubular reactor, which measures 60 cm in length, 8 mm in inner diameter, and has a wall thickness of 1.5 mm. Without any pretreatment, 150 mg of the catalyst precursor was secured in the reactor using quartz wool. Subsequently, the CO_2_ hydrogenation reaction was conducted under a pressure of 1 MPa, using a mixed gas (H_2_/CO_2_/N_2_ = 72%/24%/4%) with a weight hourly space velocity (WHSV) of 8000 mL g_cat_^−1^ h^−1^. The BVO-x samples were evaluated over a temperature range of 450 to 325 °C, and each temperature was maintained for 3 h to ensure stable isothermal activity. For the Ni/BVO-x samples, experiments were conducted at 400 °C for 3 h. Similarly, controlled experiments with Bi-based or V-based catalysts, as well as HCl post-treated catalysts, were also carried out at 400 °C for 3 h. The quantities of Bi-based or V-based catalysts used in these experiments are detailed in Supplementary Table 4. Cycle experiments were carried out at 400 °C for 3 h, followed by cooling to room temperature to complete one cycle. This procedure was repeated three times. The reaction products were continuously analyzed using a gas chromatographic system (GC-9160) equipped with both a flame ionization detector (FID) and a thermal conductivity detector (TCD). This setup allowed for the monitoring of concentrations of CO_2_, CO, CH_4_, and CH_3_OH. Catalytic performance was assessed based on CO_2_ conversion and the selectivity toward three products: CH_4_, CO, and CH_3_OH. Catalytic performance was calculated using the following equations:[6]

CO_2_ conversion (%) = $\frac{\left[ \text{CO} \right]+\left[ \text{C}\text{H}_{4} \right]+\left[ \text{C}\text{H}_{3}\text{OH} \right]}{\left[ \text{CO} \right]+\left[ \text{C}\text{H}_{4} \right]+\left[ \text{C}\text{H}_{3}\text{OH} \right]+\left[ \text{C}\text{O}_{2} \right]}\text{×100\%}$ (1)

CO selectivity (%) = $\frac{\left[ \text{CO} \right]}{\left[ \text{CO} \right]+\left[ \text{C}\text{H}_{4} \right]+\left[ \text{C}\text{H}_{3}\text{OH} \right]}\text{×100\%}$ (2)

CH_4_ selectivity (%) = $\frac{\left[ \text{C}\text{H}_{4} \right]}{\left[ \text{CO} \right]+\left[ \text{C}\text{H}_{4} \right]+\left[ \text{C}\text{H}_{3}\text{OH} \right]}\text{×100\%}$ (3)

CH_3_OH selectivity (%) = $\frac{\left[ \text{C}\text{H}_{3}\text{OH} \right]}{\left[ \text{CO} \right]+\left[ \text{C}\text{H}_{4} \right]+\left[ \text{C}\text{H}_{3}\text{OH} \right]}\text{×100\%}$ (4)

where [CO], [CH_4_], [CH_3_OH]and [CO_2_] were the outlet of CO, CH_4_, CH_3_OH and CO_2_ in the reactor, respectively.

## In situ DRIFTS measurement

In situ DRIFTS measurements were conducted using a Thermo Scientific Nicolet iS50 spectrometer equipped with a liquid nitrogen-cooled Mercury-Cadmium-Telluride (MCT) detector in the scanning wavenumber range from 4000 to 650 cm^−1^. 10 mg of the sample was placed in the infrared cell (Harrick) equipped with a ZnSe window. Spectra (64 scans) were collected at a resolution of 4 cm^−1^ and processed using the Nicolet OMNIC software. Prior to each measurement, the samples underwent H_2_ pretreatment (15 mL min^−1^) at 400 °C for 3 h, followed by N_2_ purging (20 mL min^−1^) for 1 h. The in situ DRIFT spectra were recorded under three distinct test conditions after the pretreatment. (1) CO_2_ hydrogenation at 0.1 MPa: the sample was first treated under a CO_2_ flow (5 mL min^−1^) at 400 °C for 30 min, and the gas was then switched to H_2_ flow (15 mL min^−1^) for 30 min. Finally, a CO_2_/H_2_ gas mixture (CO_2_/H_2_ = 25%/75%) was introduced at 400 °C for 30 min. (2) CO desorption: the sample was exposed to a CO flow (5 mL min^−1^) for 20 min at 40 °C, then purged with N_2_ flow (20 mL min^−1^) for 20 min to observe CO adsorption. (3) CO_2_ hydrogenation reaction at 400 °C and 1 MPa: the test was conducted under a CO_2_/H_2_ gas mixture (CO_2_/H_2_ = 25%/75%, 20 mL min^−1^) at 400 °C and 1 MPa for 30 min. Subsequently, CO_2_ gas was stopped, and the pressure was maintained at 1 MPa by flowing H_2_ (15 mL min^−1^) for 30 min.

## Mass spectrometry measurement

The CO_2_ pulse experiments, Temperature-Programmed Surface Reaction (TPSR), and H/D exchange reaction between H_2_ and D_2_ were conducted using a multifunctional adsorption instrument coupled with the OmniStar GSD320 mass spectrometer. Prior to all experiments, 150 mg of precursor was reduced in the reactor at 400 °C for 3 h under H_2_ flow (15 mL min^−1^) and then swept by He flow (20 mL min^−1^) for 30 min. To administer CO_2_ pulses, the tubular quartz reactor was maintained at 400 °C. Pure CO_2_ (0.99 mL) was pulsed into the catalysts using He (50 mL min^−1^) as the carrier gas, at least five times. Afterward, the catalysts were oxidized with pure O_2_ flow (5 mL min^−1^) for 1 h, followed by another round of CO_2_ pulse administration. Finally, after pure H_2_ reduction (15 mL min^−1^) for 1 h, CO_2_ pulses were conducted again. For CO_2_ hydrogenation Temperature-Programmed Surface Reaction (CO_2_-TPSR), after cooling to 50 °C under He flow (20 mL min^−1^), the temperature was gradually raised from 50 to 600 °C at a rate of 5 °C min^−1^ under a CO_2_/H_2_ atmosphere (H_2_/CO_2_/N_2_ = 72%/24%/4%, 20 mL min^−1^). The effluent gases were monitored by a mass spectrometer (MS, Pfeiffer Omistar GSD320) while scanning the fragments of CO (m/z = 28). For the H/D exchange experiment, the sample was then exposed to pure H_2_ for 20 min at 400 °C, after which metered pulses of high-purity D_2_ were introduced into the H_2_ stream. Signals at m/z = 2 (H_2_), 3 (HD), and 4 (D_2_) were monitored online.

## DFT theoretical calculation method

The Vienna ab initio simulation package (VASP) was used to implement DFT calculations.[7] The electron-ion interactions were treated within the projector-augmented wave (PAW) method. The generalized gradient approximation-Perdew-Burke-Ernzerhof (GGA-PBE) functional was used to describe the semilocal exchange and correlation.[8] A plane-wave cutoff energy of 500 eV was used.[9-10] The DFT-D3 correction method proposed by Grimme et al.[11] was employed in this study to consider the dispersion interaction. The ionic relaxation was allowed until the residual force on each atom was below 0.05 eV Å^–1^; the total energy difference was allowed under a threshold of 10^–5^ eV during the structure optimization. The Brillouin zone was sampled using a 2 × 2 × 1 Monkhorst-Pack *k*-point mesh.[12] The transition states were roughly identified with the climbing image-nudged elastic band method (CI-NEB) and further refined with the dimer method.[13-15] The force threshold for the determination of transition states was set to be 0.05 eV Å^–1^. Furthermore, all transition states were confirmed by only one imaginary frequency. The VASPKIT toolkit was used to obtain free energy corrections under the typical experimental conditions at 673.15 K.[16]


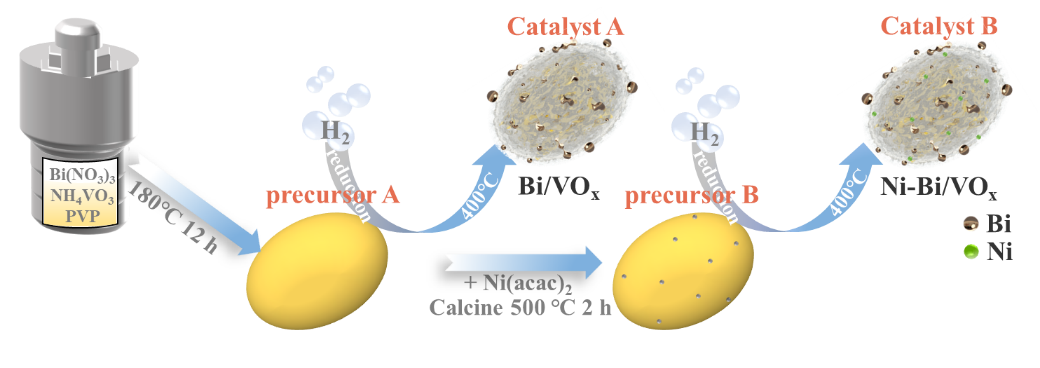


# **Figure S1.** Schematic illustration depicting the structural evolution via H_2_-mediated reduction from BiVO_4_ precursors to Bi-based catalysts.


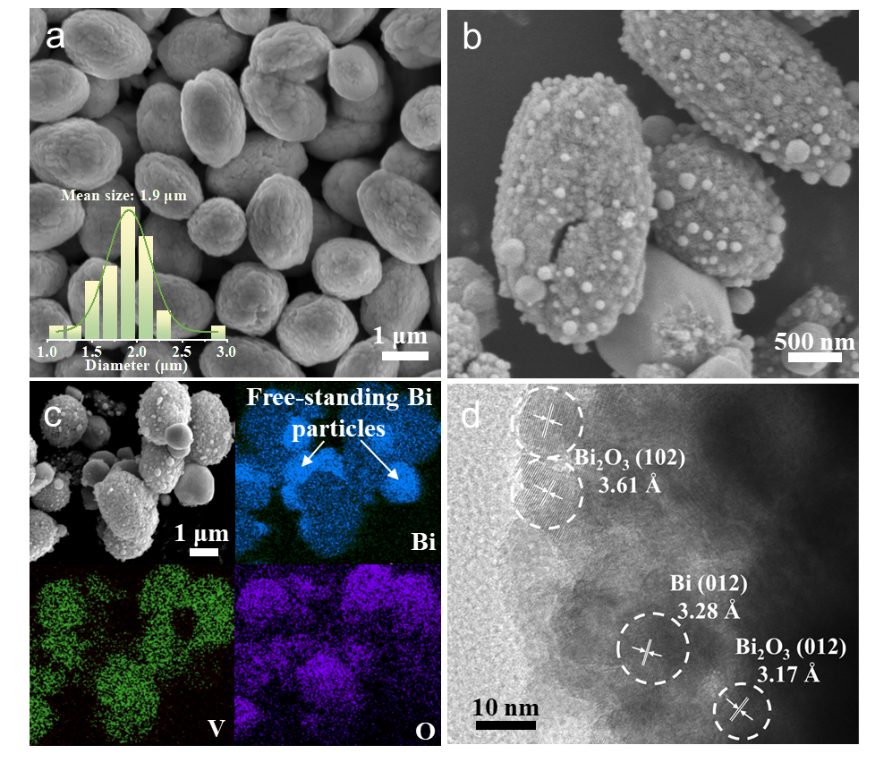


# **Figure S2.** a) SEM image of BVO precursor (Inset: size statistic of BVO particles, with an average long axis of 1.9 μm). b) SEM image of u-BVO. c) SEM image with corresponding elemental maps of u-BVO. (Arrows: free-standing Bi particles) d) HRTEM image of u-BVO (The circled areas highlight the observed lattice fringe regions).


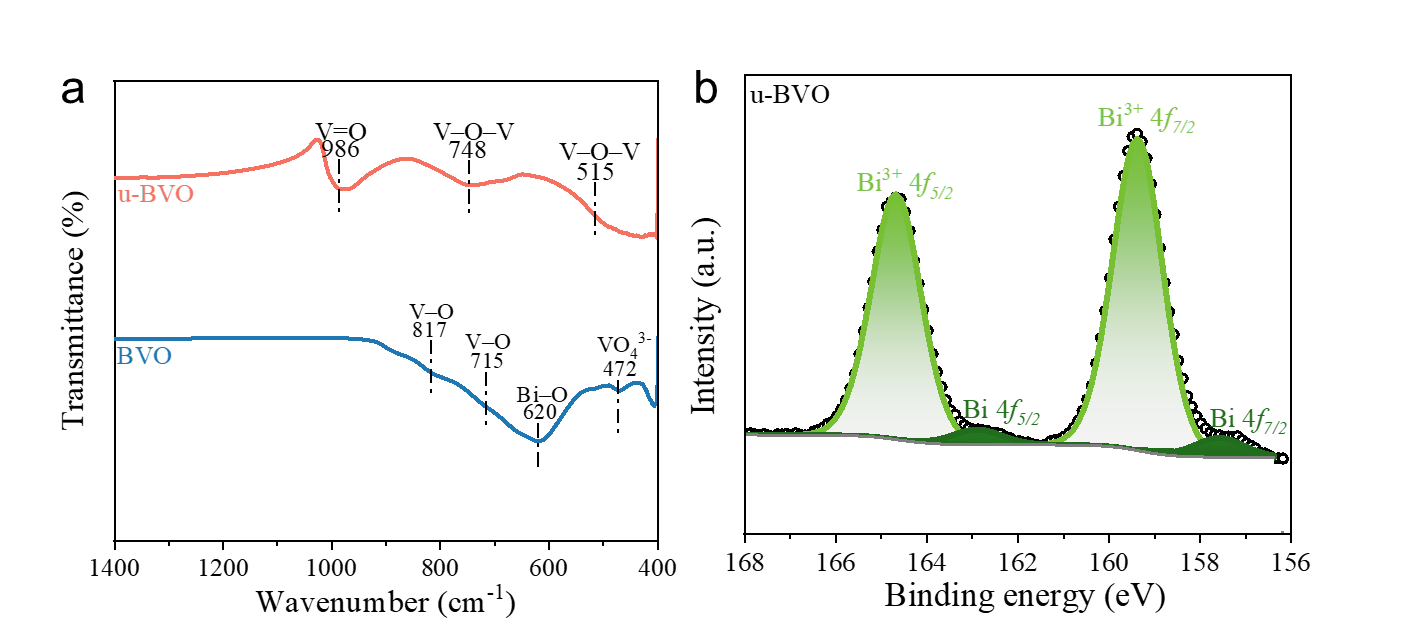


# **Figure S3.** a) FTIR spectra of BVO and u-BVO. b) XPS profiles of the Bi 4*f* peak for u-BVO.


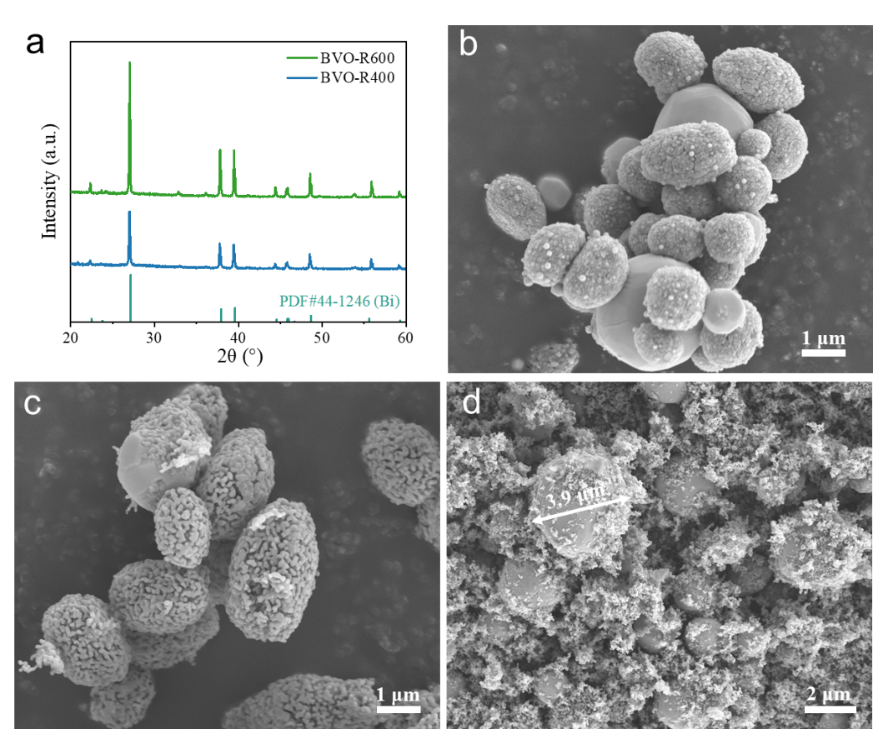


# **Figure S4.** a) XRD patterns of BVO-R600 and BVO-R400. b-d) SEM images of b) BVO-R400 and c-d) BVO-R600.


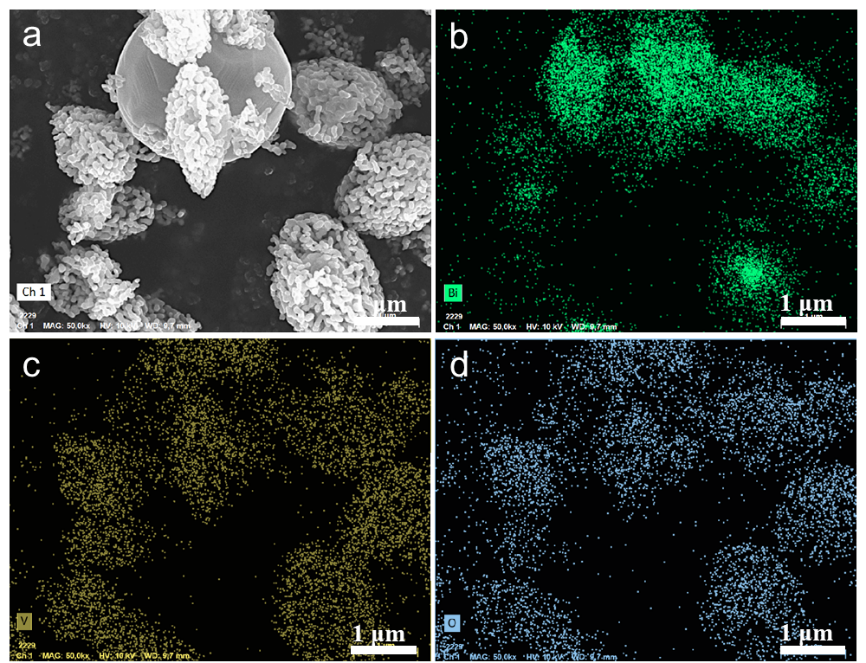


# **Figure S5.** The EDS elemental maps of BVO-R600.


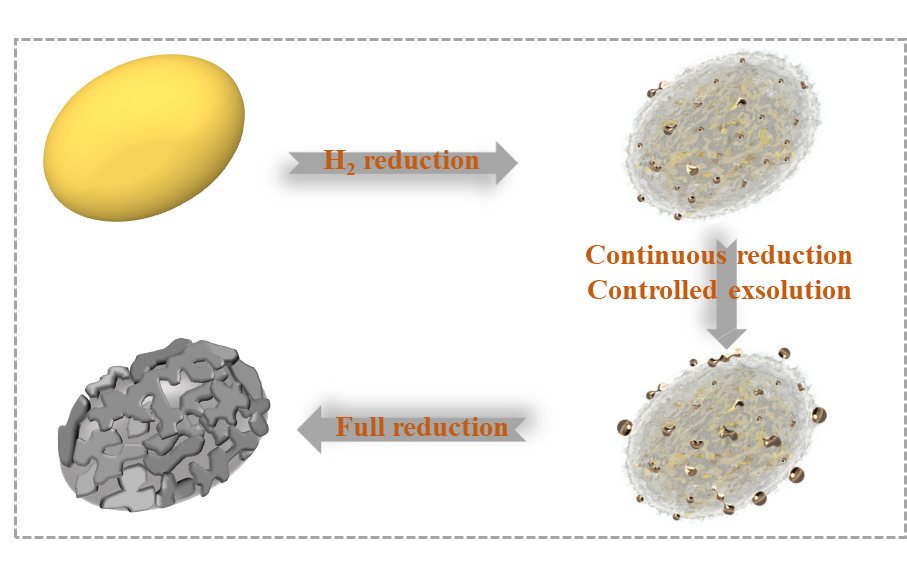


# **Figure S6.** Schematic representation of Bi/VO_x_ structure evolution derived from BiVO_4_ precursors.


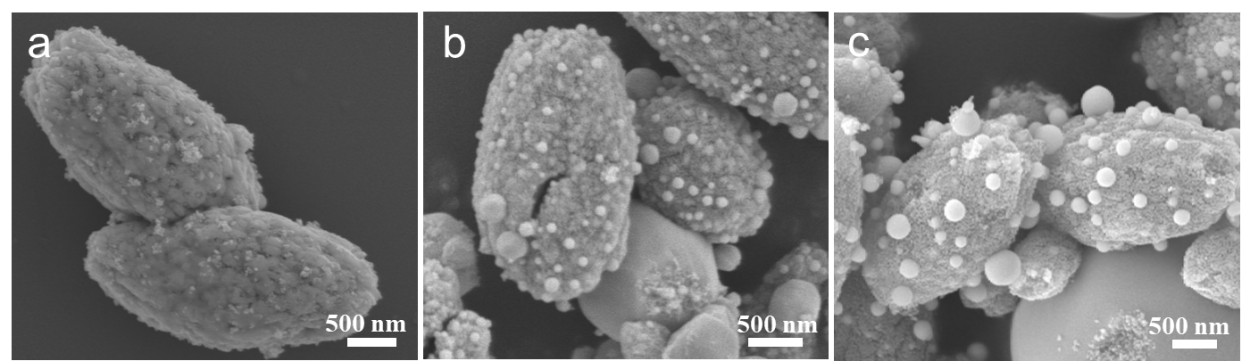


# **Figure S7.** a-c) time evolution of SEM images of u-BVO collected after reaction at 400 °C for a) 1 h, b) 3 h, and c) 50 h.


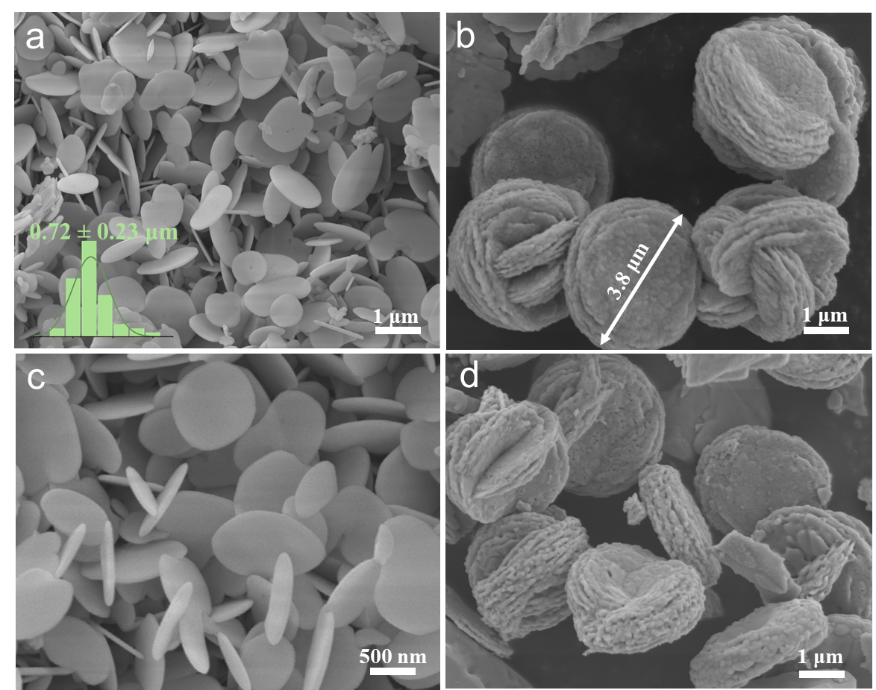


# **Figure S8.** a, c) SEM images of a) BVO-c and c) Ni/BVO-c precursors. (Inset of a: size statistic of BVO-c particles, with an average diameter of 0.72 μm). b, d) SEM images of b) BVO-a and d) Ni/BVO-a precursors.


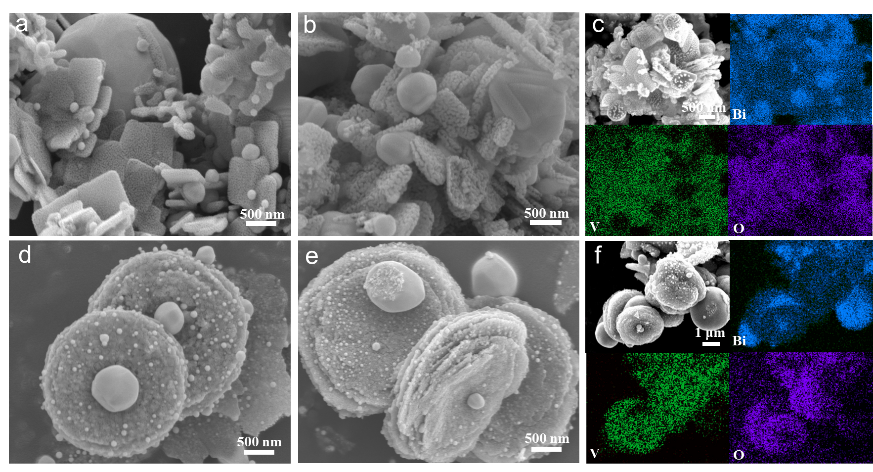


# **Figure S9.** a-b) SEM images of a) u-BVO-c and b) u-Ni/BVO-c. c) EDS elemental maps of u-BVO-c. d-e) SEM images of d) u-BVO-a and e) u-Ni/BVO-a. f) EDS elemental maps of u-BVO-a.


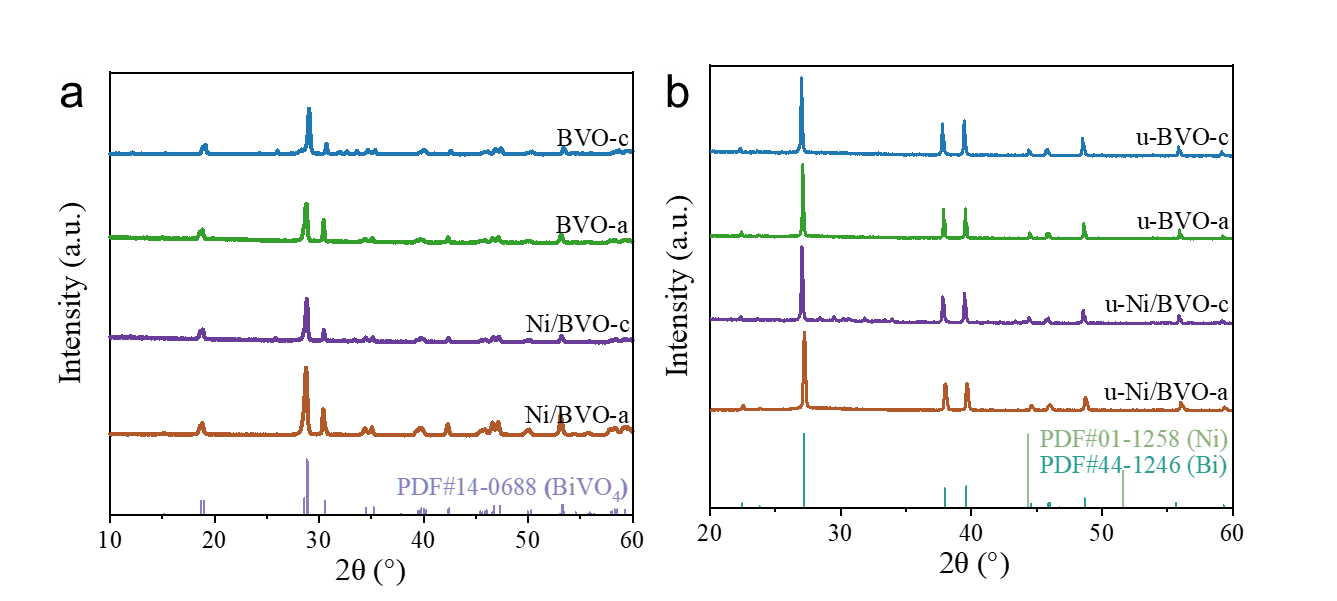


# **Figure S10.** a-b) XRD patterns of samples a) before and b) after reaction for 3 h at 400 °C.

**
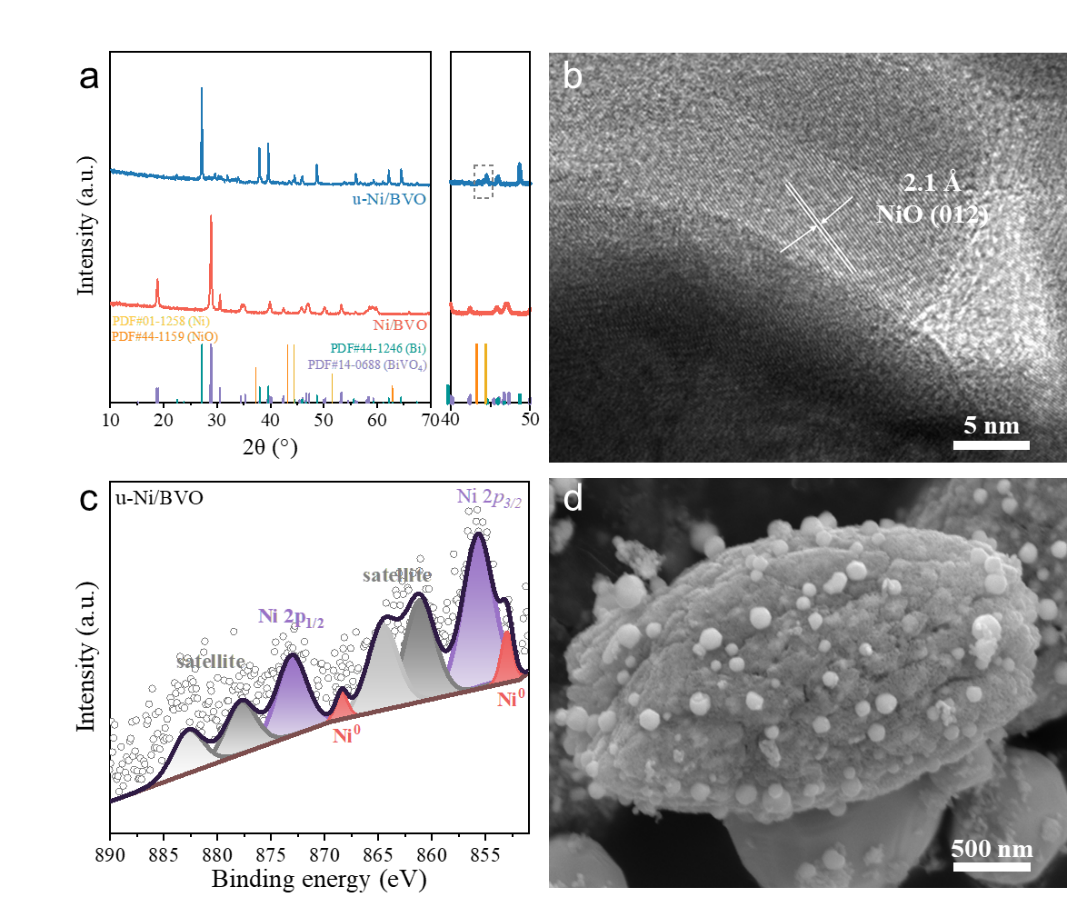
**

# **Figure S11.** a) XRD patterns of Ni/BVO and u-Ni/BVO. b) HRTEM image of u-Ni/BVO. c) XPS profile of Ni 2*p* of u-Ni/BVO. d) SEM image of u-Ni/BVO.


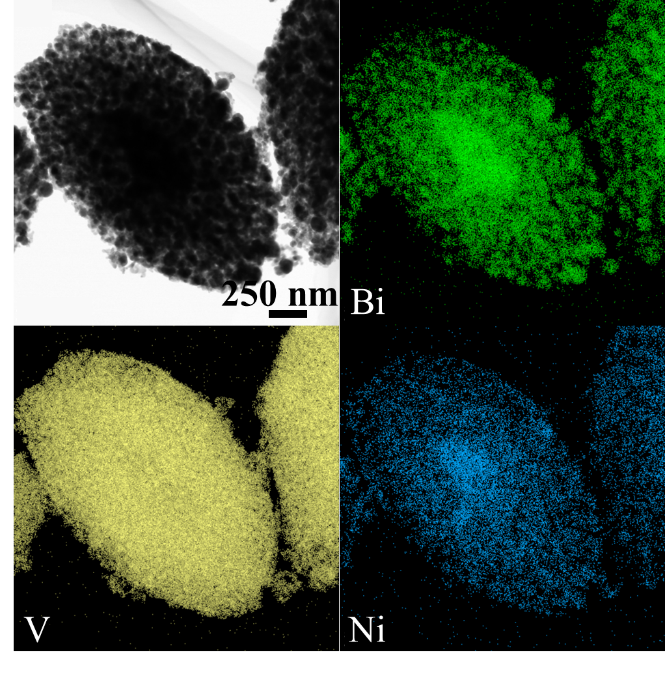


# **F****igure S12.** TEM image with corresponding EDS elemental maps of u-Ni/BVO.


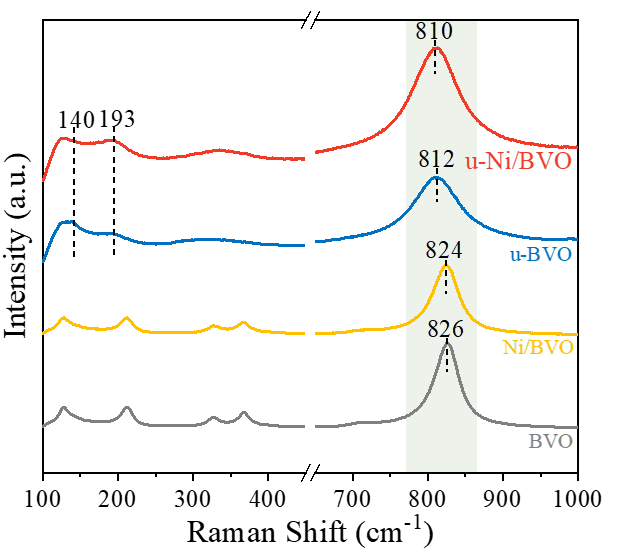


# **Figure S13.** Raman spectra of samples.

_
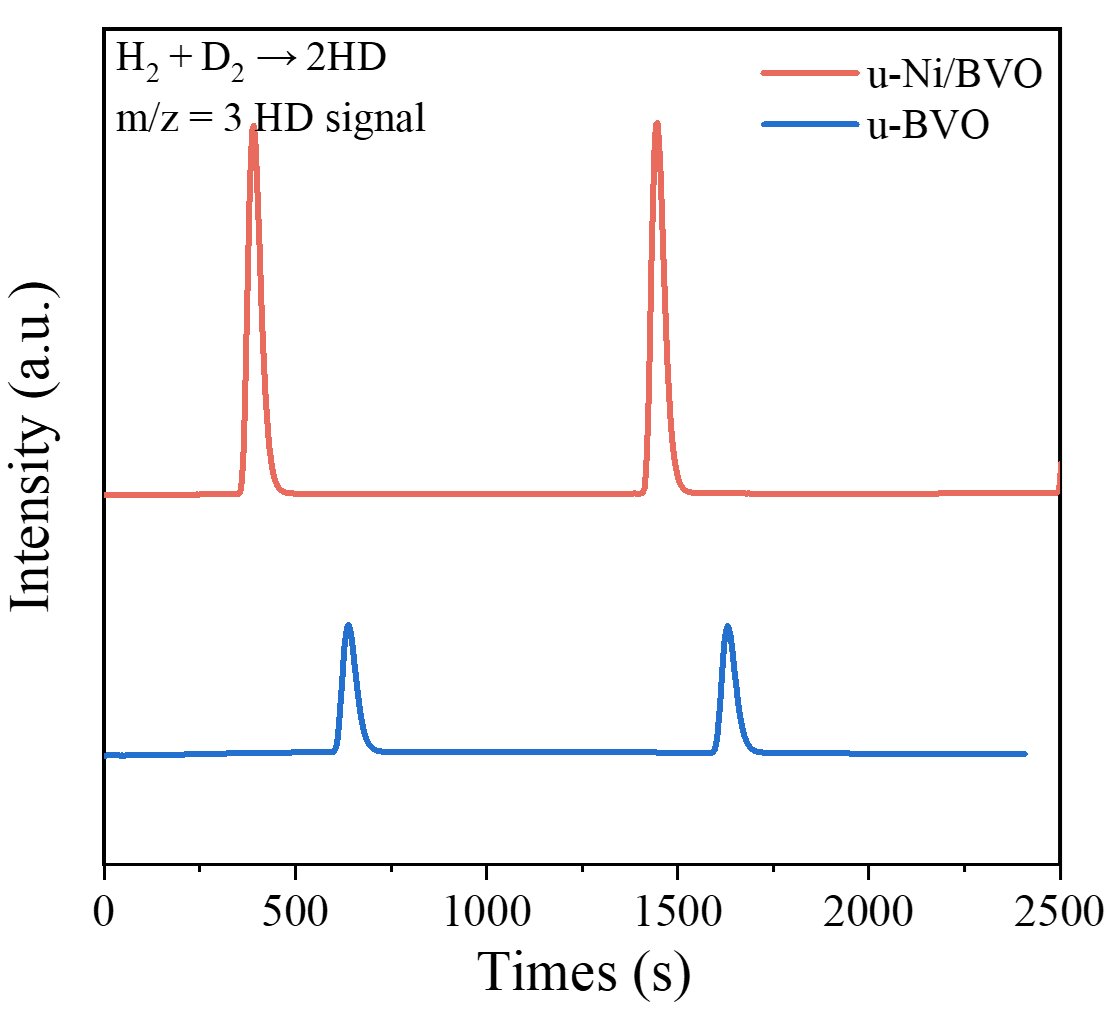
_

# **Figure S14.** HD (m/z = 3) signal intensity as a function of time during the H_2_-D_2_ exchange reaction over u-Ni/BVO and u-BVO.


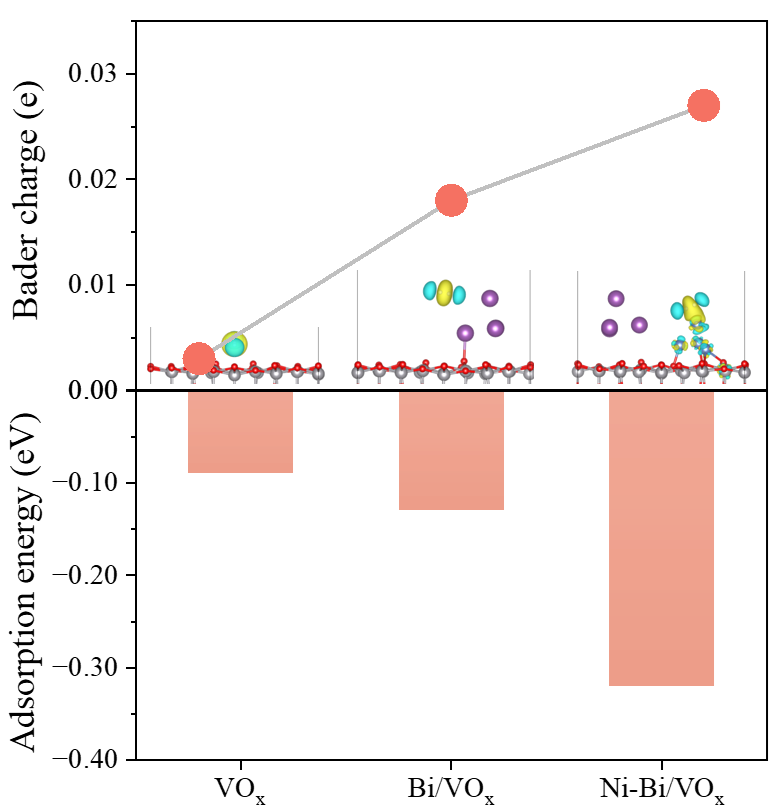


# **Figure S15.** The differential charge density, Bader charge, and adsorption free energy of H_2_ adsorbed on the VO_x_, Bi/VO_x_, and Ni-Bi/VO_x_ models, the iso-surface is 0.0004 eÅ^-3^.


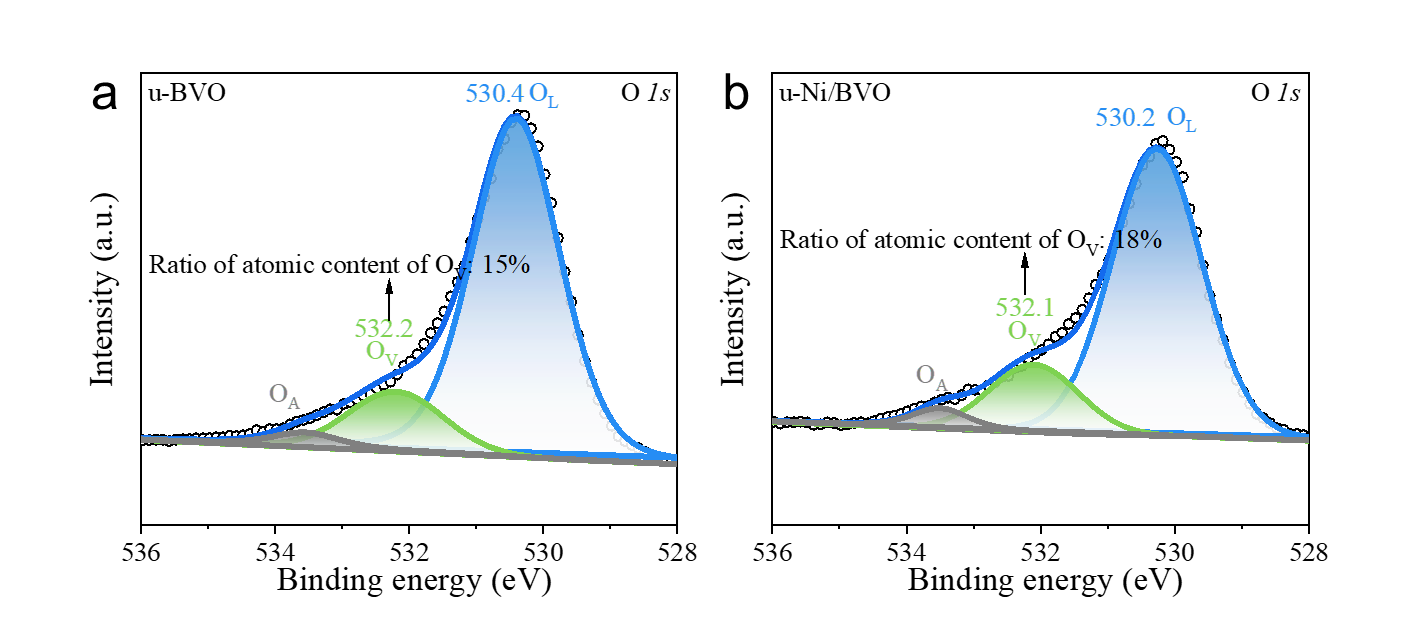


# **Figure S16.** a-b) XPS profiles of O 1*s* of a) u-BVO and b) u-Ni/BVO.


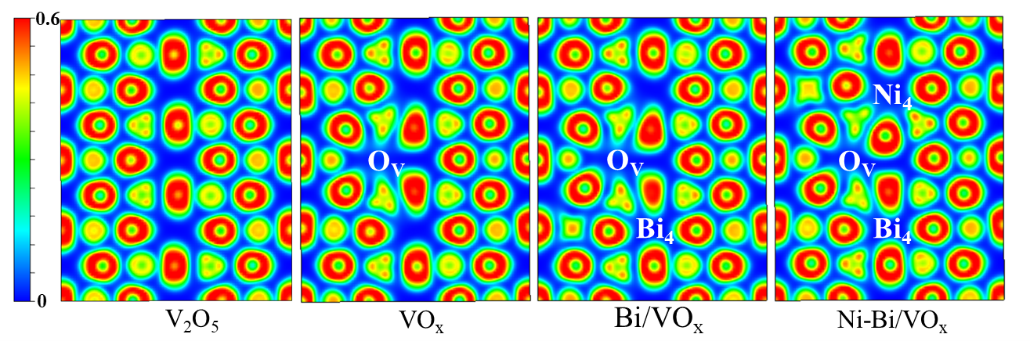


# **Figure S17.** The electron localization function of V_2_O_5_, VO_x_, Bi/VO_x_, and Ni-Bi/VO_x_ models.


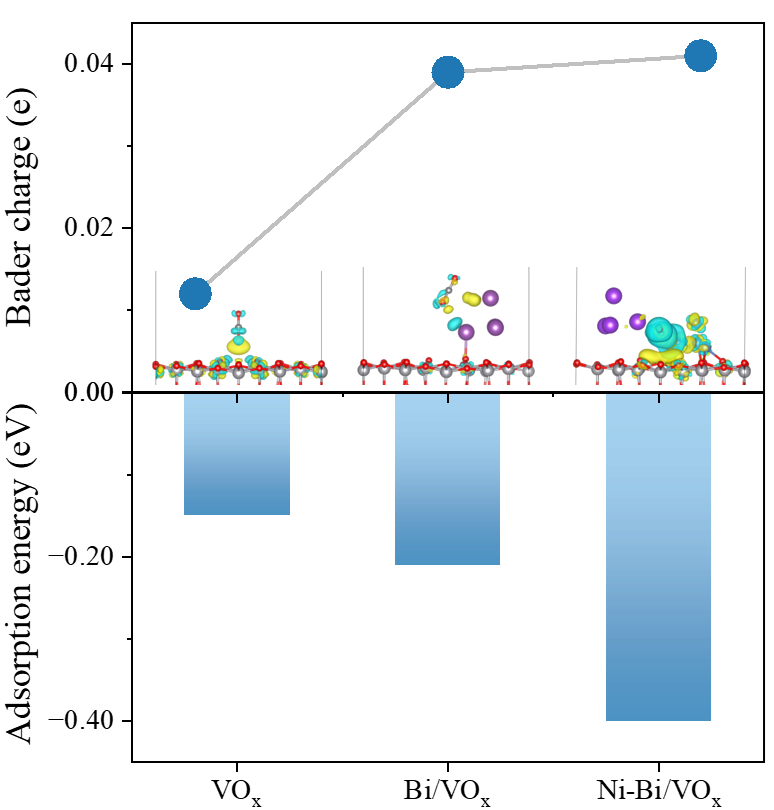


# **Figure S18.** The differential charge density, Bader charge, and adsorption free energy of CO_2_ on the VO_x_, Bi/VO_x_, and Ni-Bi/VO_x_ models, the iso-surface is 0.0004 eÅ^-3^.


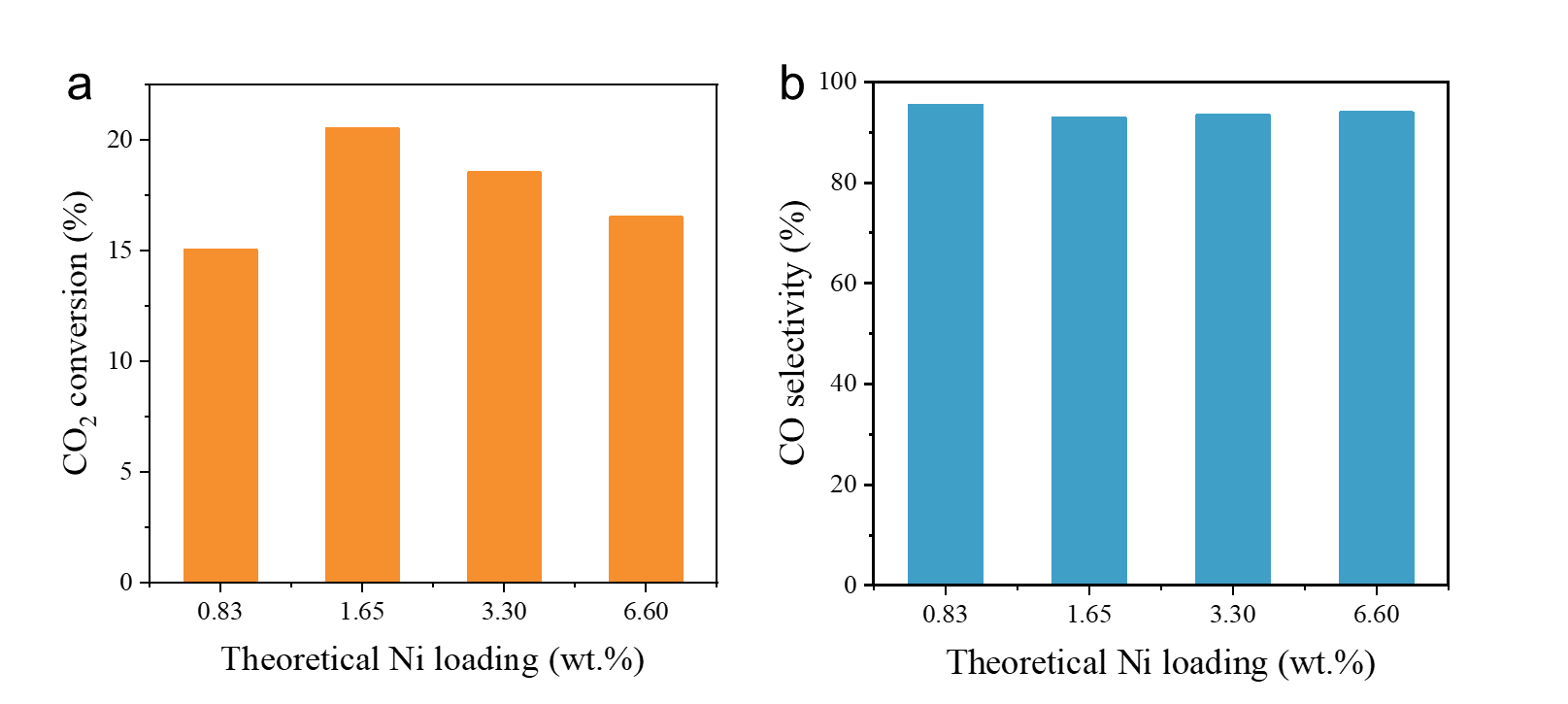


# **Figure S19**. a) CO_2_ conversion and b) CO selectivity of u-Ni/BVO catalysts with different theoretical Ni loading (wt.%).


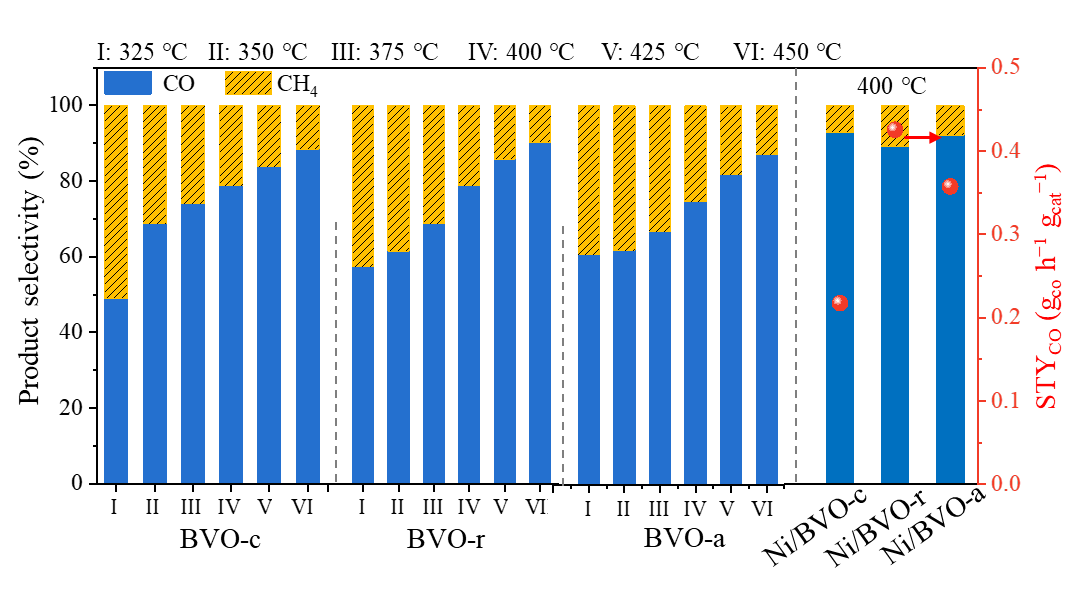


# **Figure S20.** Product selectivity and STY of CO for the as-prepared catalysts with different morphologies.

**
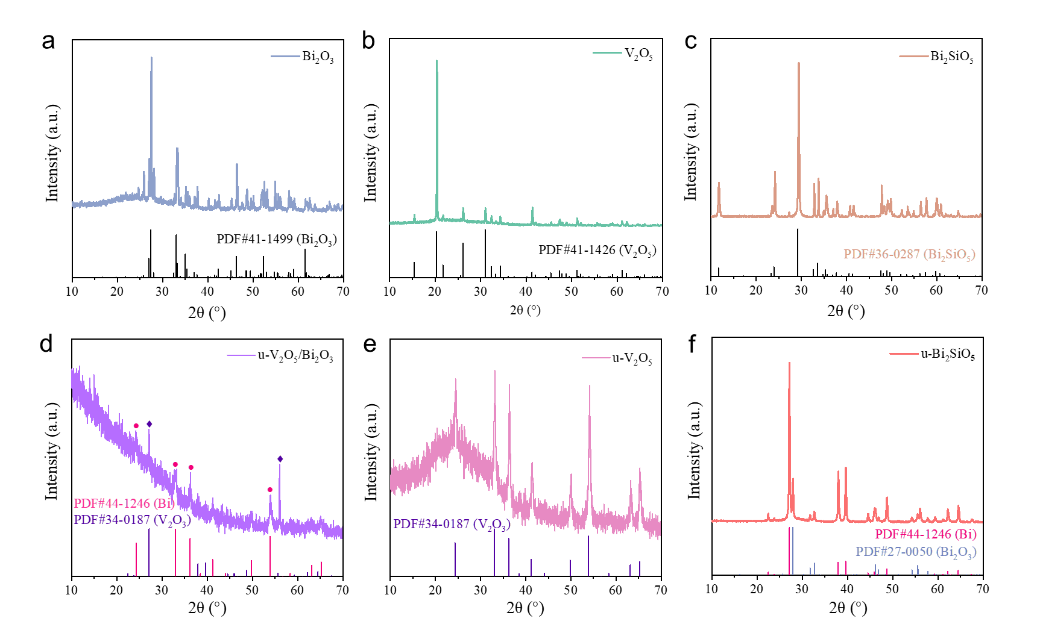
**

# **Figure S21.** a-f) XRD patterns of a) Bi_2_O_3_, b) V_2_O_5_ & c) Bi_2_SiO_5_ precursors, and d) u-Bi_2_O_3_ + V_2_O_5_, e) u-V_2_O_5_, and f) u-Bi_2_SiO_5_ samples collected after CO_2_ hydrogenation for 3 h at 400 °C.


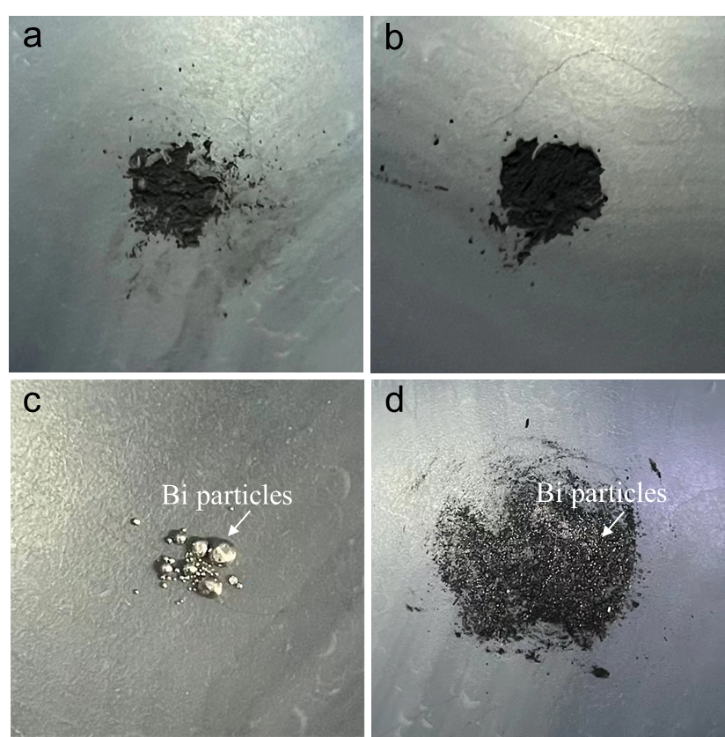


# **Figure S22.** a-d) digital photos of the a) u-BVO, b) u-V_2_O_5_, c) u-Bi_2_O_3_ (Arrow: Bi particles), and d) u-Bi_2_O_3_ + V_2_O_5_ (Arrow: Bi particles), which were collected after CO_2_ hydrogenation for 3 h at 400 °C.


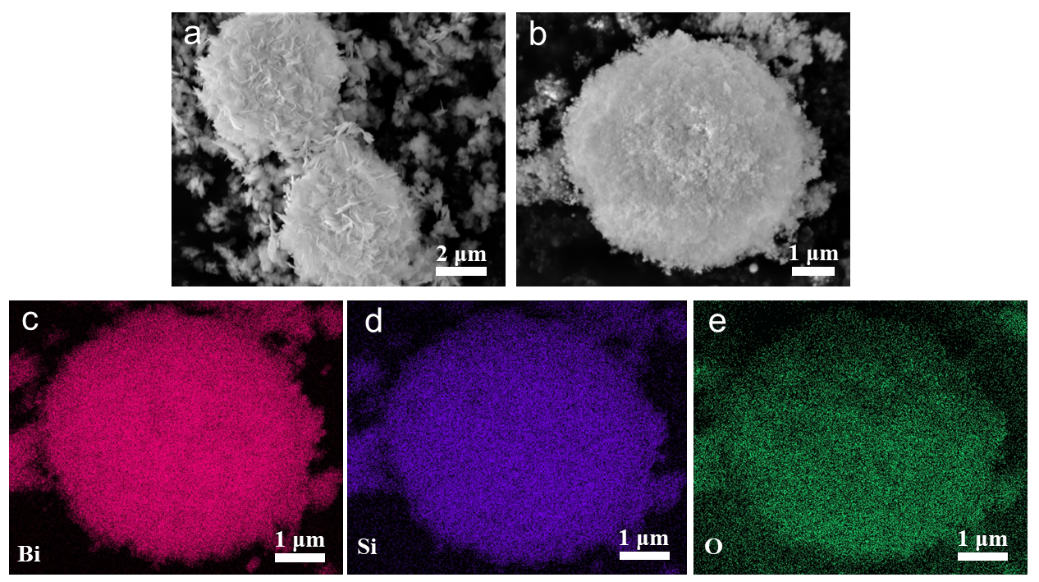


# **Figure S23.** a) SEM image of Bi_2_SiO_5_ precursor. b-e) SEM image with corresponding elemental maps of u-Bi_2_SiO_5_ collected after CO_2_ hydrogenation for 3 h at 400 °C.


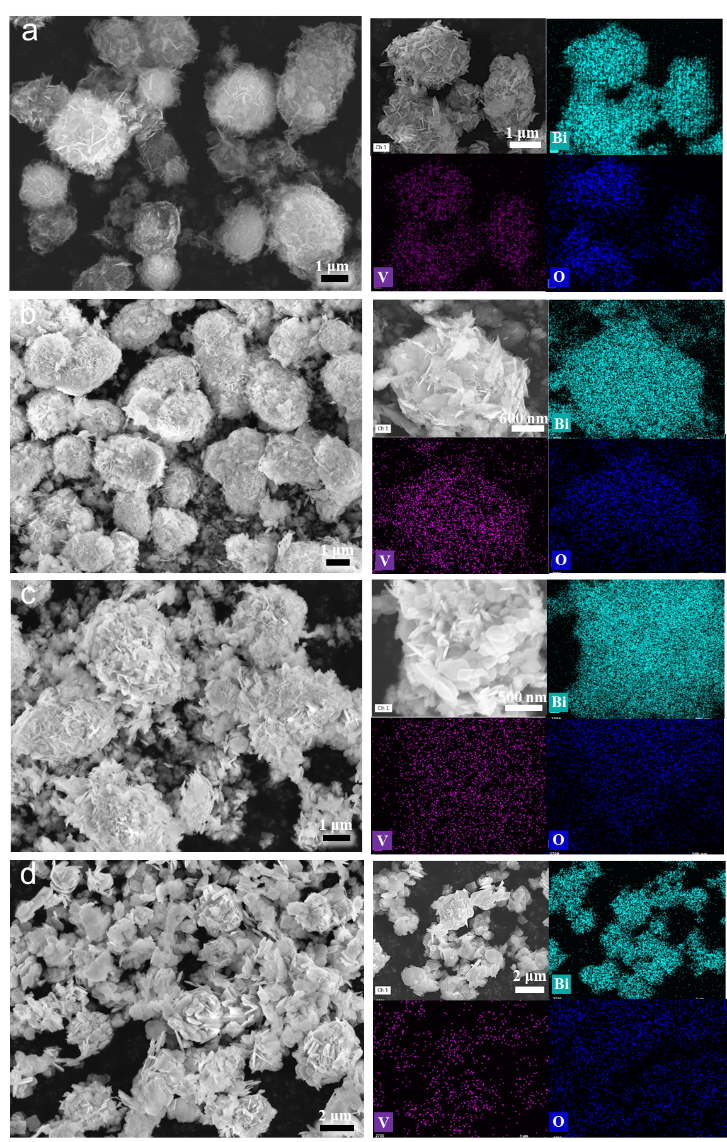


# **Figure S24.** a-d) SEM images with corresponding elemental maps of u-BVO etched by different concentrations of HCl, separately as a) 0.03, b) 0.05, c) 0.1, and d) 0.3 M.

**
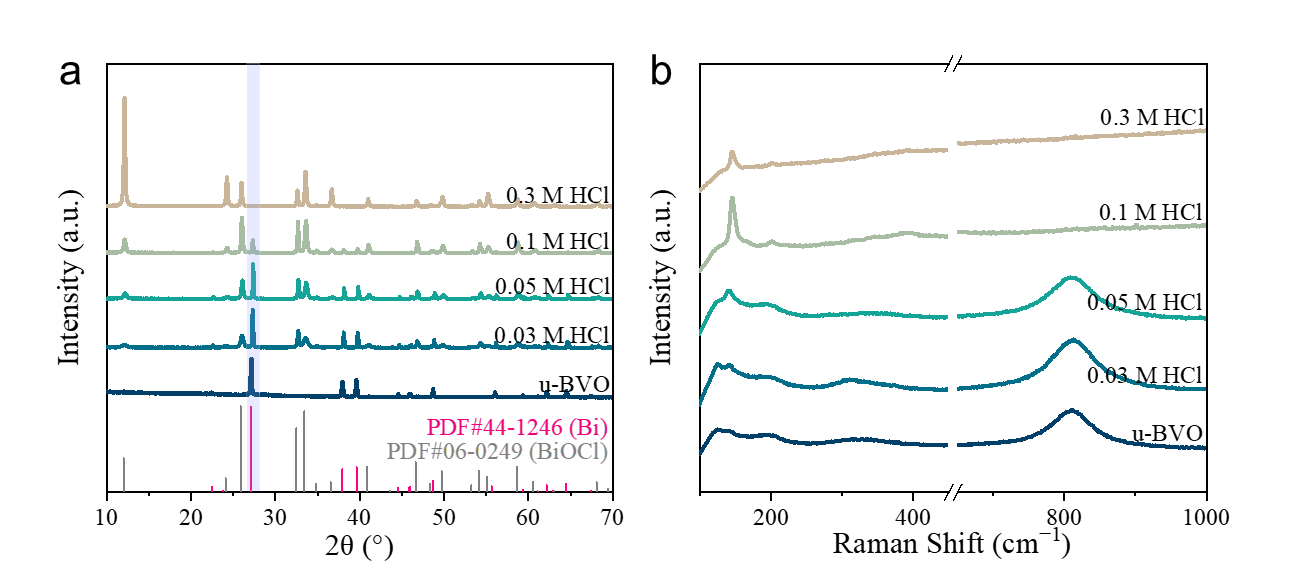
**

# **Figure S25.** a) XRD patterns, and b) Raman spectra of u-BVO etched by different concentrations of HCl.


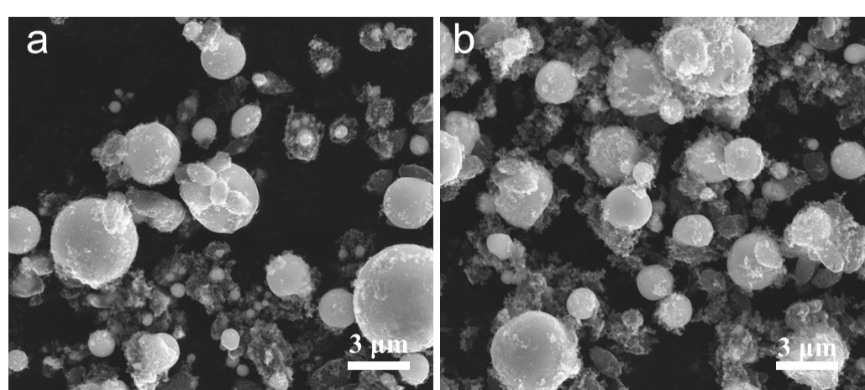


# **Figure S26.** a-b) SEM images of used a) u-BVO_0.03_ and b) u-BVO_0.05_, which were collected after CO_2_ hydrogenation for 3 h at 400 °C.

**
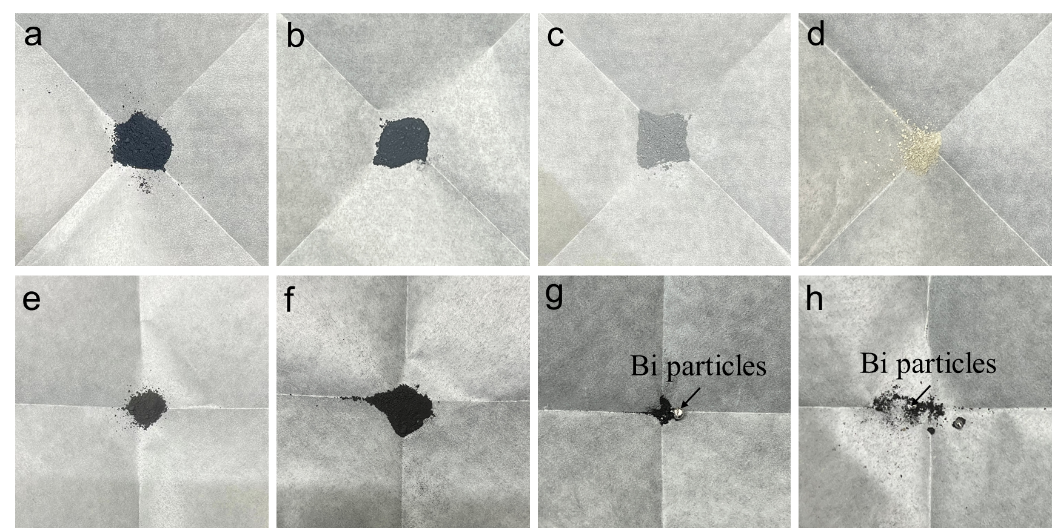
**

# **Figure S27.** a-h) digital photos of u-BVO after a) 0.03, b) 0.05, c) 0.1, & d) 0.3 M HCl etchant, and e) u-BVO_0.01_, f) u-BVO_0.05_, g) u-BVO_0.1_, & h) u-BVO_0.3_ samples, which were collected after CO_2_ hydrogenation for 3 h at 400 °C.


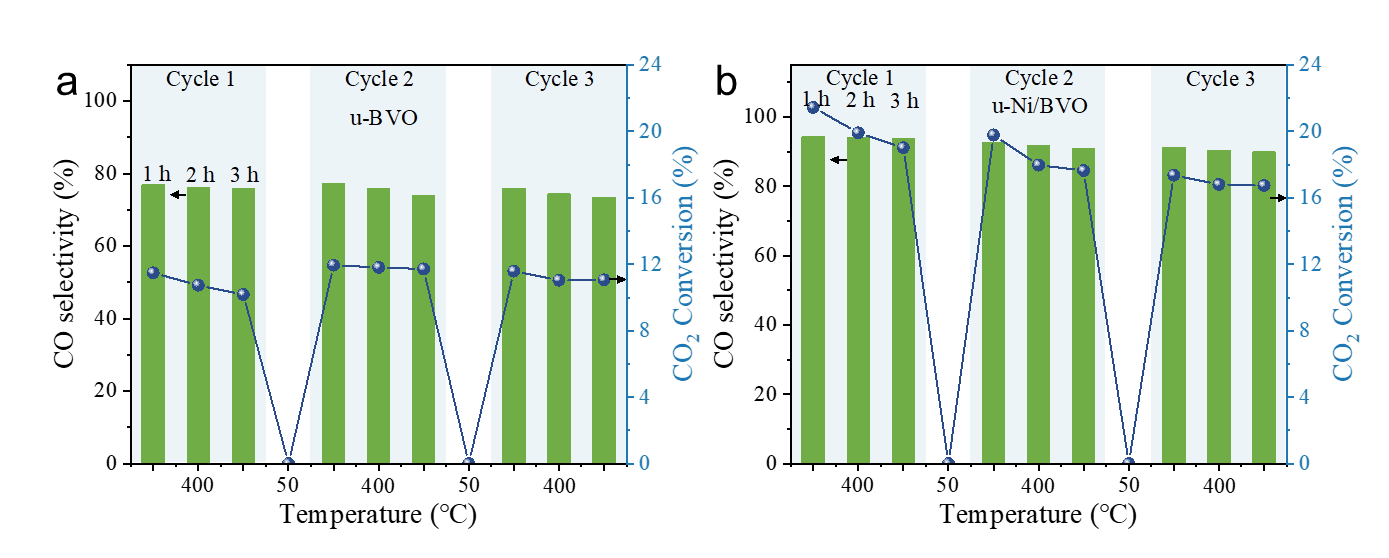


# **Figure S28.** a-b) the cycle experiments of reaction (400 °C)-cooling (50 °C) over a) u-BVO and b) u-Ni/BVO.

**
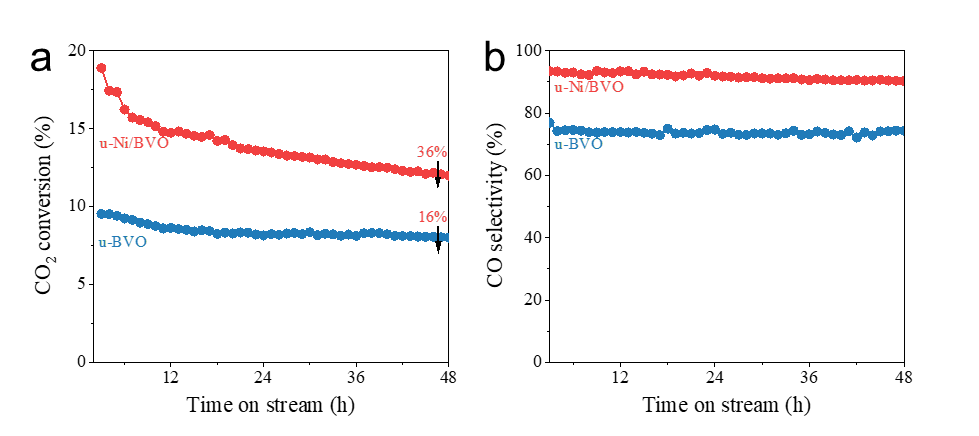
**

# **Figure S29**. a) CO_2_ conversion and b) CO selectivity on u-BVO and u-Ni/BVO during long-term stability test.


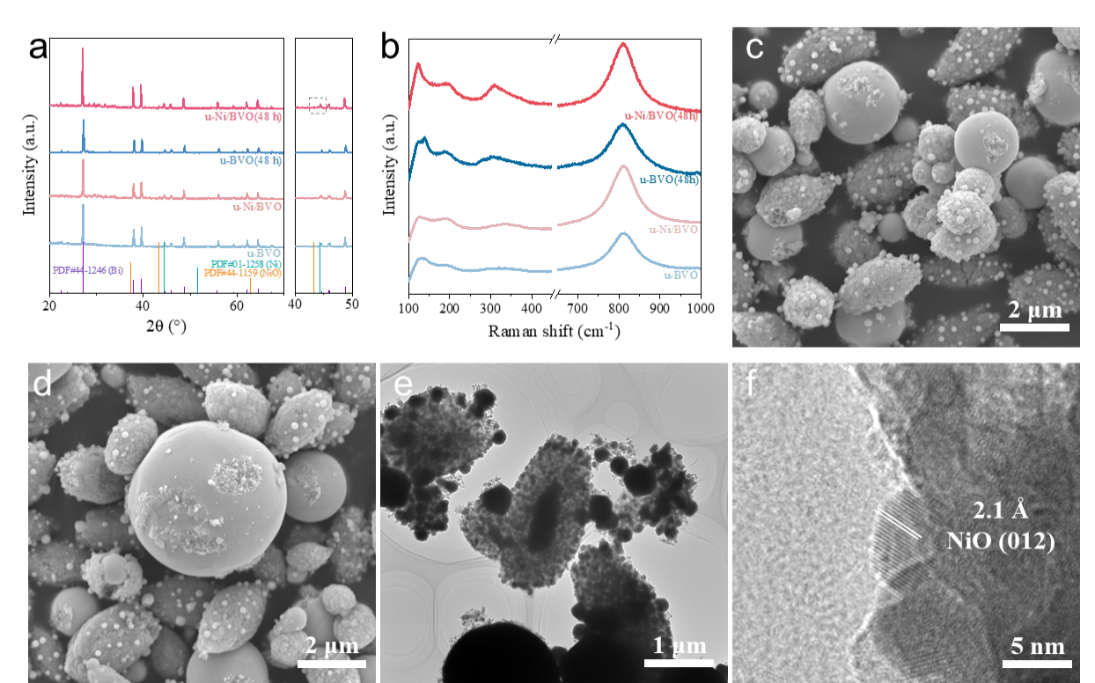


**Figure S30**. a) XRD patterns of samples. b) Raman spectra of samples. c-d) SEM images of c) u-BVO(48 h) and d) u-Ni/BVO(48 h). e) TEM and f) HRTEM images of u-Ni/BVO(48 h) collected after CO_2_ hydrogenation for 48 h at 400 °C.


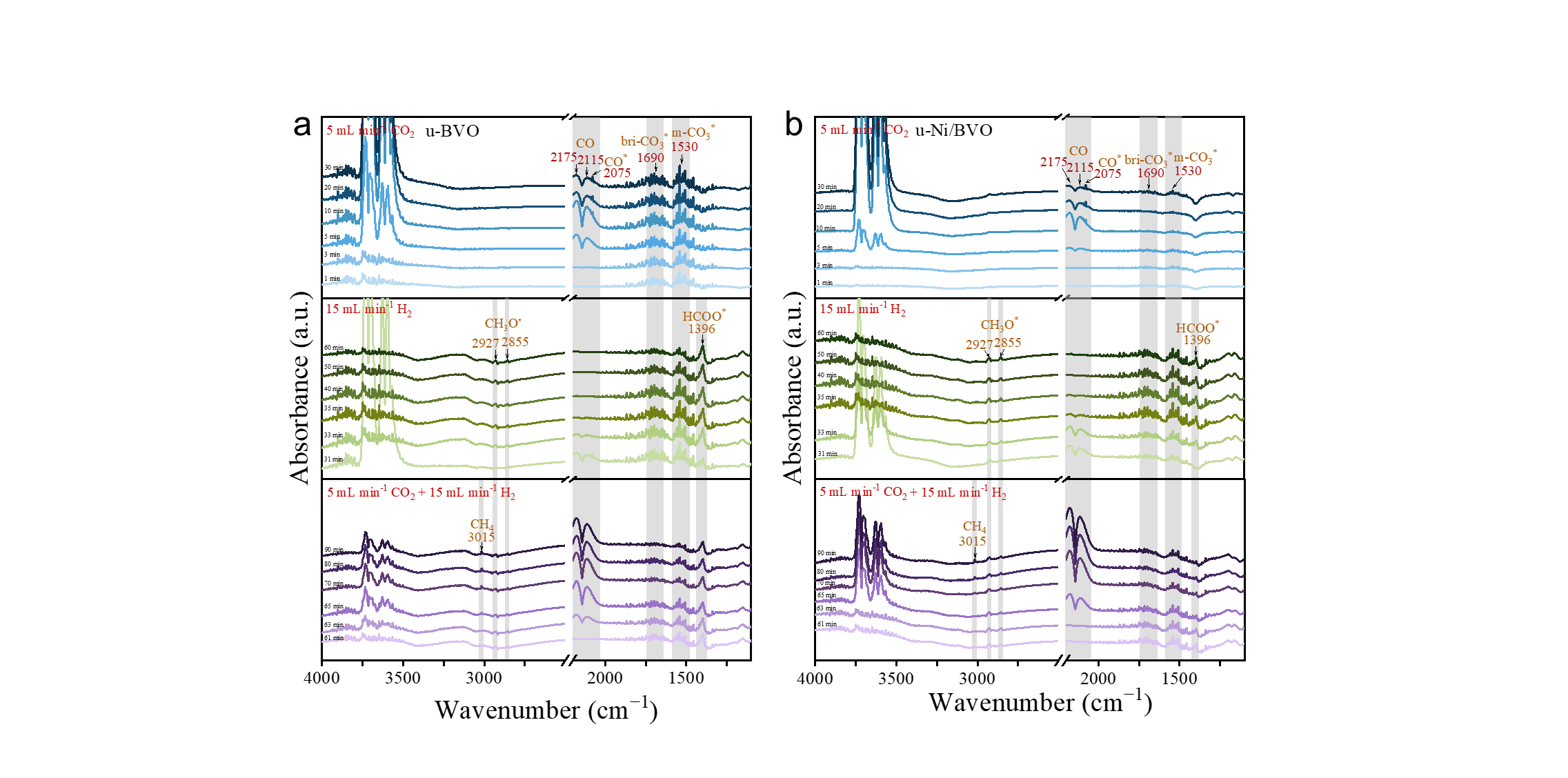


# **Figure S31.** a-b) in situ DRIFTS analysis of a) u-BVO, and b) u-Ni/BVO at 400 °C and 0.1 MPa.


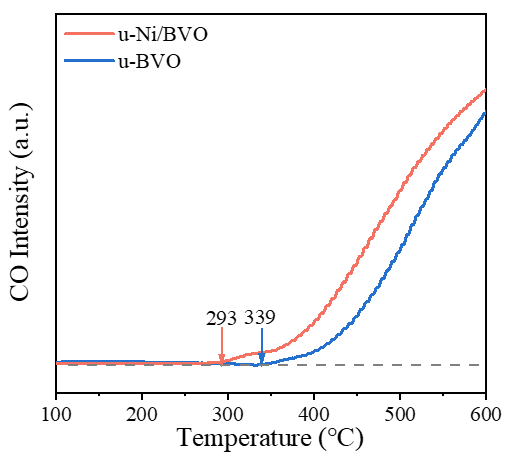


# **Figure S32.** TPSR curves of u-BVO and u-Ni/BVO.


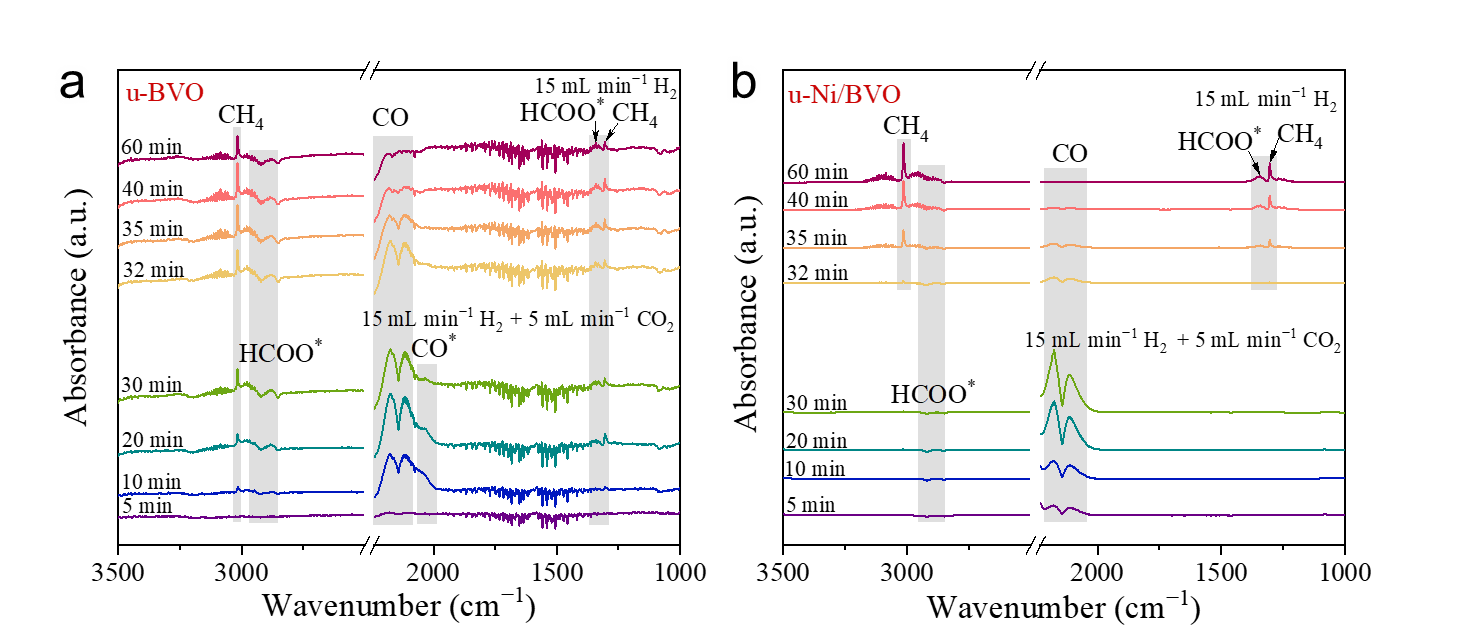


# **Figure S33.** a-b) in-situ DRIFTS analysis of a) u-BVO, and b) u-Ni/BVO at 400 °C and 1 MPa.


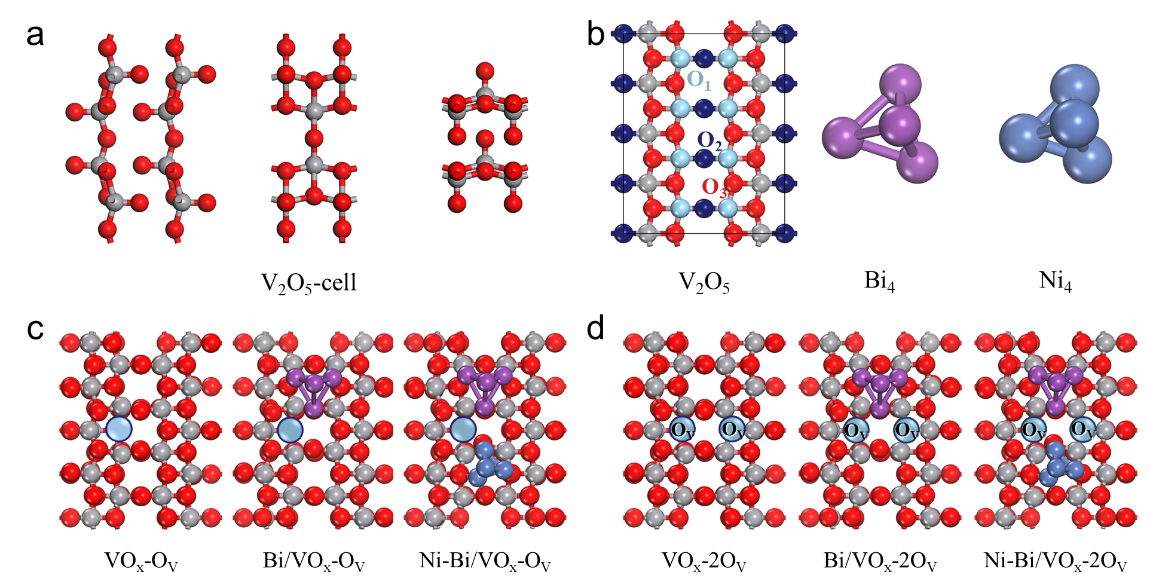


# **Figure S34.** a) the crystal cell of V_2_O_5_. b) the type of oxygen vacancies (O_V_) on the surface of V_2_O_5,_ and the loaded metal clusters (Bi_4_ cluster and Ni_4_ cluster). c-d) three different catalyst models constructed c) with one oxygen vacancy and d) with two oxygen vacancies, including VO_x_, Bi/VO_x,_ and Ni/VO_x_. (Red: O, grey: V, purple: Bi, blue: Ni)

Notes: The static Bi_4_ cluster serves as a targeted computational model designed to probe the electronic structure of low-coordination surface atoms, which represent the predominant active sites on the surfaces of liquid Bi nanodroplets. This approach does not aim to simulate the entire droplet, but rather to capture the key electronic features of its catalytically relevant regions. The strategy is justified by the well-documented atomic-scale layering and short-range order of liquid metal surfaces, which confer transient local structural rigidity.[17-19] Consequently, the cluster provides a representative electronic “snapshot” of the dynamic catalytic interface.

The periodic V_2_O_5_ (001) model is employed as a computationally tractable proxy for the amorphous VO_x_ support. While it simplifies the long-range structural disorder, it retains the essential local [VOₓ] coordination units and the metal-support interfacial chemistry that govern catalytic behavior.[20-21] This model enables the decoding of fundamental electronic-structure trends (such as charge transfer, oxygen vacancy energetics, and adsorbate binding strengths) whose predicted behaviors align consistently with experimental observations, thereby offering a coherent mechanistic basis for the catalytic performance.

The *α*-V_2_O_5_ crystal phase and (001) surface of V_2_O_5_ were determined, with the cell-optimized lattice constants *α* = *β* = *γ* = 90°, a = 11.64 Å, b = 3.61 Å, and c = 4.33 Å (as shown in Fig. S29a).[22-23] V_2_O_5_(001) contains three types of lattice oxygens: mono-coordinated terminal oxygens O_1_, bi-coordinated O_2_ and tri-coordinated oxygens O_3_. The terminal O (O_1V_) is unstable under reaction conditions (a hydrogen-rich atmosphere) and is easily carried away; thus, the formation of oxygen vacancies on V_2_O_5_(001) is considered for O_2V_ and O_3V_. O_3V_ is more easily formed, as indicated by the lower formation energy (*E*_O3V_ = 3.21 eV) compared to O_2V_ (*E*_O2V_ = 3.67 eV). For the second oxygen vacancy (2O_V_), the oxygen vacancy formation energy (*E*_2OV_) is in the order: Bi/VO_x_-2O_V_ (2.89 eV) < Ni/VO_x_-2O_V_ (3.16 eV) < VO_x_-2O_V_ (3.24 eV).


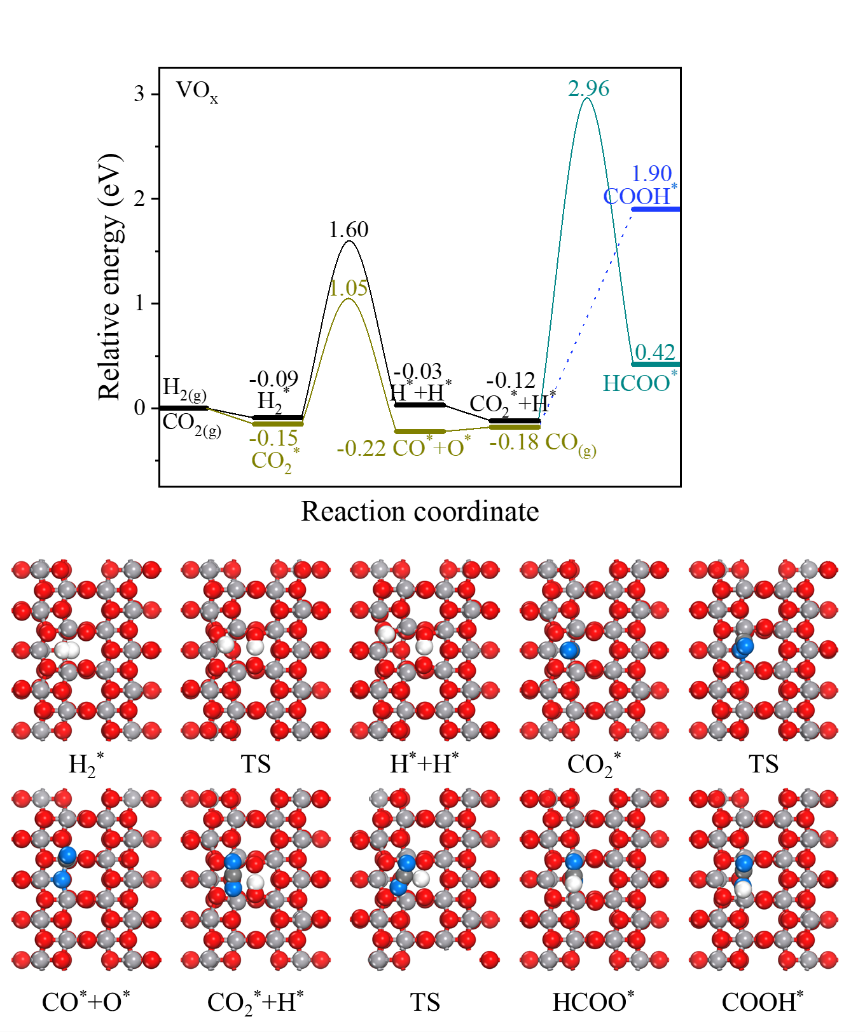


# **Figure S35.** Relative energy profile of CO_2_ and H_2_ activation over the VO_x_ model, as well as the structures of the initial state, transition state, and final state.


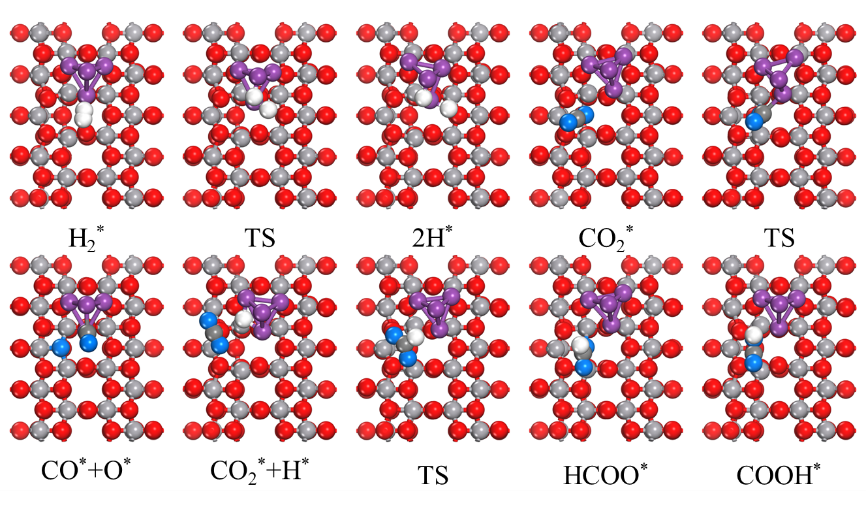


# **Figure S36.** The structures of the initial state, transition state, and final state for CO_2_ and H_2_ activation over the Bi/VO_x_ model.


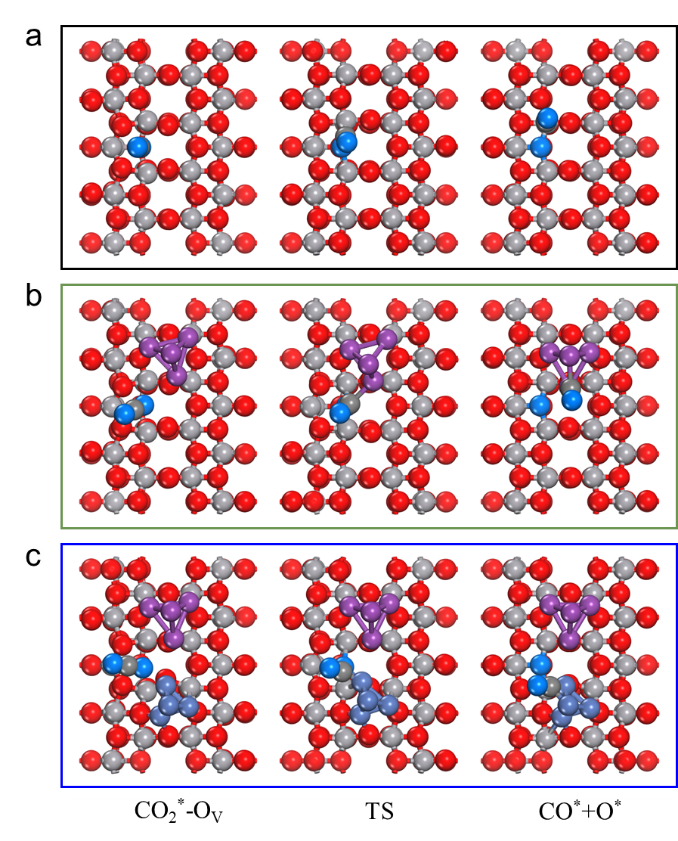


# **Figure S37.** a-c) the structures of the initial state, transition state, and final state of CO_2_ adsorption and direct dissociation to form CO and O at O_V_ site over a) VO_x_, b) Bi/VO_x_, and c) Bi-Ni/VO_x_ models.

Notes: The calculated trend of weak CO^*^ adsorption, rooted in the distinct electronic structure of the Bi sites, provides a direct and consistent electronic-structure rationale for the high CO selectivity observed experimentally. Together, these findings establish a robust, multiscale evidence chain: macroscopic catalytic tests confirm the active role of Bi⁰ nanodroplets, while atomic-scale DFT calculations elucidate the mechanistic origin of the observed selectivity at their surface sites.


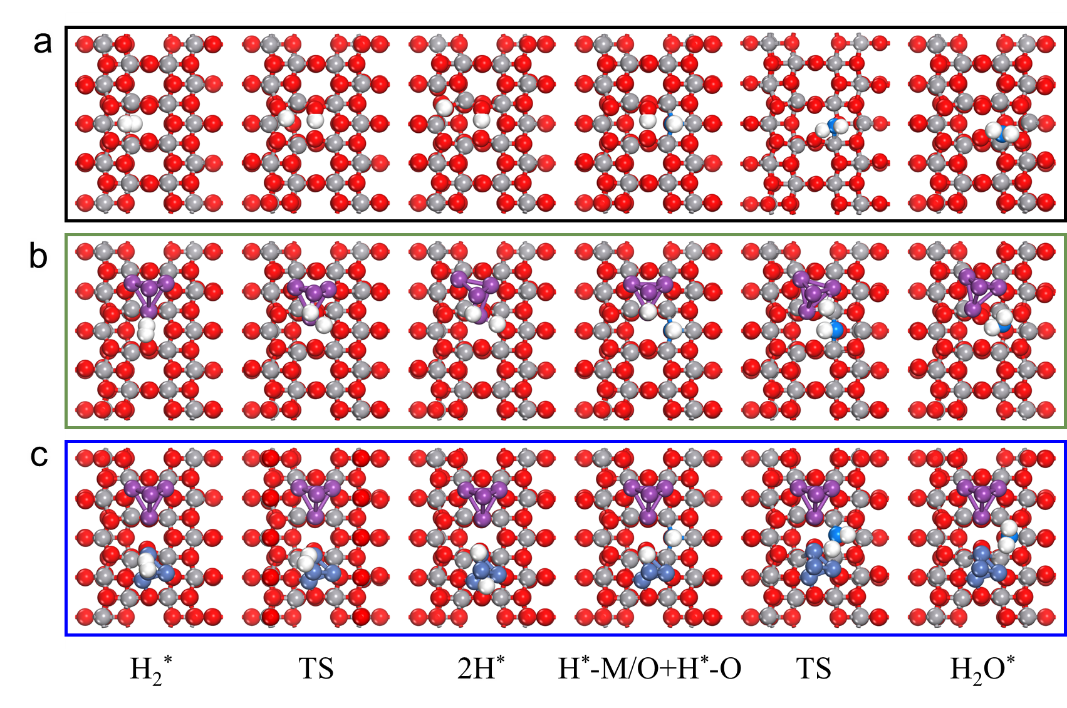


# **Figure S38.** a-c) the structures of the initial state, transition state, and final state of H_2_ dissociation and O_V_ regeneration over a) VO_x_, b) Bi/VO_x_, and c) Bi-Ni/VO_x_ models.


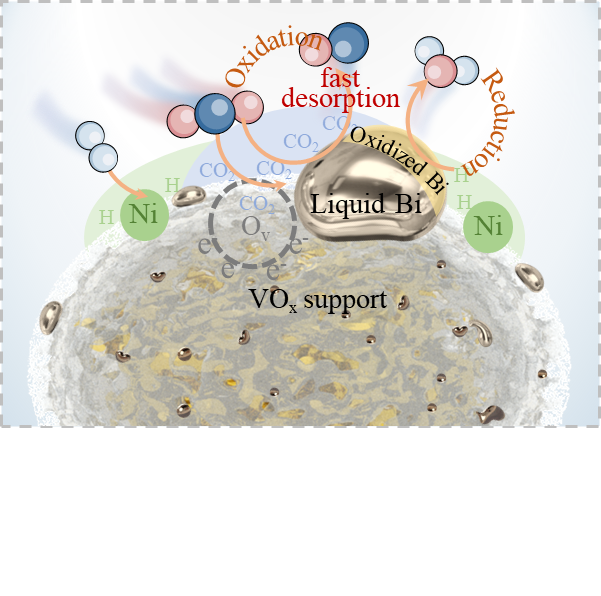


# **Figure S39.** The schematic illustration of the redox mechanism on the Ni-Bi/VO_x_ surface.

# **Table S1.** List of Bi, V, and Ni contents of Ni/BVO-x quantified by ICP-OES.

| Sample | Bi (wt.%) | V (wt.%) | Ni (wt.%) |
| --- | --- | --- | --- |
| Ni/BVO-c | 68.6 | 11.7 | 1.9 |
| Ni/BVO-r | 73.4 | 16.7 | 1.6 |
| Ni/BVO-a | 64.7 | 13.5 | 1.4 |

# **Table S2.** List of recently reported high-performance catalysts for CO_2_ selective hydrogenation to CO.

| Catalyst | Metal content (wt.%) | H_2_/CO_2_/N_2_  (%) | Temp.  (°C) | Press.  (MPa) | GHSV  (mL g_cat_^−1^ h^−1^) | C_CO2_  (%) | S_CO_  (%) | Ref. |
| --- | --- | --- | --- | --- | --- | --- | --- | --- |
| Bi/VO_x_ | Bi-73.4 | 72/24/4 | 400 | 1 | 8000 | 10 | 75 | This work |
| Ni-Bi/VO_x_ | Bi-73.4 / Ni-1.6 |  |  |  | 8000 | 20 | 90 |  |
| Pb-Bi/CeO_2_ | Pd-2.0 | 73/24/3 | 240 | 3 | 2000 | ~5 | 74 | [24] |
| Ni-Ce(OH)_3_/CeO_2_ | 0.5 | 25/8.3/66.6 | 400 | 0.1 | 72,000 | ~28 | 100 | [25] |
| NiWC | 2.0 | 60/20/20 | 400 | 0.1 | 12000 | ~5 | 100 | [26] |
| NiTe/TiO_2_ | Ni-1.9 / Te-3.9 | 60/20/20 | 400 | 4.0 | 15000 | ~30 | 100 | [27] |
| Ni^𝛿+^−PSNS | N/A | 45/45/10 | 300 | 0.1 | 10000 | 11 | 92 | [28] |
| PdNi/SiO_2_ | N/A | 25/25/50 | 400 | 0.1 | 600000 | ~2 | 100 | [29] |
| Ni/CeZrO_2_ | 1.0 | H_2_:CO_2_=1:1 | 550 | 0.1 | 10000 | ~34 | 54 | [30] |
| Ni/nSiO_2_ | 2.4 | 40/10/50 | 400 | 0.1 | 400000 | ~38 | ~80 | [31] |
| NiMo/SiO_2_ | Ni-5 / Mo-8.2 | 40/10/50 | 400 | 0.1 | 100000 | 24 | 94 | [32] |
| Ni/Al_2_O_3_ | 0.4 | H_2_:CO_2_=1:1^a^ | 400 | 0.1 | 200000 | ~8 | 100 | [33] |
| NiIn/Al_2_O_3_ | Ni-43.3 / In-42.3 | H_2_:CO_2_=4:1 | 400 | 0.1 | 30000 h^-1^ | ~30 | 100 | [34] |
| NiCu/SiO_2_ | Ni-9.0 / Cu-1.0 | 20/5/75 | 400 | 0.1 | 60000 | ~33 | ~74 | [35] |
| Ni/CNTs | 2.2 | H_2_:CO_2_=3:1 | 400 | 3 | 12000 | ~18 | 99 | [36] |
| Ni/CeO_2_ | 1.1 | 40/10/50 | 300 | 0.1 | 18000 | ~10 | 100 | [37] |
| NiCu/Sap | Ni-13.9 / Cu-13.5 | H_2_:CO_2_=4:1 | 400 | 0.1 | 15000 | ~18 | 100 | [38] |
| Cu/CeO_x_-MgO | 4.6 | 72/24/4 | 400 | 0.1 | 300000 | ~20 | 100 | [39] |
| Pt-CeO_2_@SiO_2_ | 0.3 | 45/15/40 | 400 | 0.1 | 12000 | 38.2 | ~100 | [40] |

^a^ CO_2_ and H_2_ concentrations were diluted to 0.1 mol mol^−1^ with Ar.

^b^ Saponite: a smectite clay (NaMg_6_(Si_7_Al)O_20_(OH)_4_)

# **Table S3.** List of grain sizes (*D*s) of catalysts calculated by the Scherrer formula using the FWHM of the strongest XRD peak.

| Samples | 2θ (°) | FWHM (°) | *D*_S_ (nm) |
| --- | --- | --- | --- |
| u-BVO-c | 27.17 | 0.13 | 60.8 |
| u-Ni/BVO-c | 27.17 | 0.14 | 56.9 |
| u-BVO-r | 27.17 | 0.17 | 47.9 |
| u-Ni/BVO-r | 27.17 | 0.17 | 47.0 |
| u-BVO-a | 27.17 | 0.17 | 47.8 |
| u-Ni/BVO-a | 27.17 | 0.18 | 45.2 |
| u-BVO-r(48 h) | 27.17 | 0.18 | 45.2 |
| u-Ni/BVO-r(48 h) | 27.17 | 0.14 | 58.2 |

# **Table S4.** List of usage amounts of Bi-based or V-based precursors.

| Catalyst precursor | Quality (mg)^a^ |
| --- | --- |
| Bi_2_O_3_ | 108 |
| Bi_2_SiO_3_ | 1225 |
| V_2_O_5_ | 42 |
| Bi_2_O_3_ + V_2_O_5_^b^ | 108 + 42 |
| Bi_2_SiO_5_ + V_2_O_5_^b^ | 1225 + 42 |

a. The amount of each element in the Bi-based or V-based precursors was the same as that in 150 mg of BVO.

b. These samples were obtained by physically mixing the corresponding precursors.

**References**

1. G. Xi, J. Ye, "Synthesis of Bismuth Vanadate Nanoplates with Exposed {001} Facets and Enhanced Visible-Light Photocatalytic Properties," *Chemical Communications* 46,·no.·11 (2010): 1893-1895, <https://doi.org/10.1039/b923435g>.

2. L. Huang, Z. Duan, Y. Song, et al., "BiVO_4_ Microplates with Oxygen Vacancies Decorated with Metallic Cu and Bi Nanoparticles for CO_2_ Photoreduction," *ACS Applied Nano Materials* 4,·no.·4 (2021): 3576-3585, <https://doi.org/10.1021/acsanm.1c00115>.

3. L. Chen, D. Meng, X. Wu, et al., "Shape-Controlled Synthesis of Novel Self-Assembled BiVO_4_ Hierarchical Structures with Enhanced Visible Light Photocatalytic Performances," *Materials Letters* 176, (2016): 143-146, <https://doi.org/10.1016/j.matlet.2016.04.112>.

4. H. Jiang, H. Dai, X. Meng, et al., "Hydrothermal Fabrication and Visible-Light-Driven Photocatalytic Properties of Bismuth Vanadate with Multiple Morphologies and/or Porous Structures for Methyl Orange Degradation," *Journal of Environmental Sciences* 24,·no.·3 (2012): 449-457, <https://doi.org/10.1016/s1001-0742(11)60793-6>.

5. Y. Chang, X. Guan, Q. Zheng, et al., "Hydrothermal Preparation of 3D Flower-Spherical Bi_2_SiO_5_ for Photocatalytic Esterification of Oleic Acid," *Chemical Industry and Engineering Progress* 41,·no.·8 (2022): 4181-4191, <https://doi.org/10.16085/j.issn.1000-6613.2021-2161>.

6. J. Guo, P. N. Duchesne, L. Wang, et al., "High-Performance, Scalable, and Low-Cost Copper Hydroxyapatite for Photothermal CO_2_ Reduction," *ACS Catalysis* 10,·no.·22 (2020): 13668-13681, <https://doi.org/10.1021/acscatal.0c03806>.

7. G. Kresse, J. Furthmiiller, "Efficiency of Ab-Initio Total Energy Calculations for Metals and Semiconductors using a Plane-Wave Basis Set," *Computational Materials Science* 6, (1996): 15-50, <https://doi.org/10.1016/0927-0256(96)00008-0>.

8. John P. Perdew, Kieron Burke, M. Ernzerhof, "Generalized Gradient Approximation Made Simple," *Physical Review Letters* 77,·no.·18 (1996): 3865-3868, <https://doi.org/10.1103/physrevlett.77.3865>.

9. P. E. Blochl, "Projector Augmented-Wave Method," *Physical Review B* 50,·no.·24 (1994): 17953-17979, <https://doi.org/10.1103/physrevb.50.17953>.

10. G. Kresse, D. Joubert, "From Ultrasoft Pseudopotentials to the Projector Augmented-Wave Method," *Physical Review B* 59,·no.·3 (1999): 1758-1775, <https://doi.org/10.1103/PhysRevB.59.1758>.

11. S. Grimme, J. Antony, S. Ehrlich, et al., "A Consistent and Accurate Ab Initio Parametrization of Density Functional Dispersion Correction (DFT-D) for the 94 Elements H-Pu," *The Journal of Chemical Physics* 132,·no.·15 (2010): 154104, <https://doi.org/10.1063/1.3382344>.

12. H. J. Monkhorst, J. D. Pack, "Special Points for Brillouin-Zone Integrations," *Physical Review B* 13,·no.·12 (1976): 5188-5192, <https://doi.org/10.1103/PhysRevB.13.5188>.

13. G. Henkelman, B. P. Uberuaga, H. Jónsson, "A Climbing Image Nudged Elastic Band Method for Finding Saddle Points and Minimum Energy Paths," *The Journal of Chemical Physics* 113,·no.·22 (2000): 9901-9904, <https://doi.org/10.1063/1.1329672>.

14. G. Henkelman, H. Jónsson, "Improved Tangent Estimate in the Nudged Elastic Band Method for Finding Minimum Energy Paths and Saddle Points," *The Journal of Chemical Physics* 113,·no.·22 (2000): 9978-9985, <https://doi.org/10.1063/1.1323224>.

15. H. Jónsson, G. Henkelman, "A Dimer Method for Finding Saddle Points on High Dimensional Potential Surfaces using Only First Derivatives," *The Journal of Chemical Physics* 111,·no.·15 (1999): 7010-7022, <https://doi.org/10.1063/1.480097>.

16. B. Huang, R. R. Rao, S. You, et al., "Cation- and pH-Dependent Hydrogen Evolution and Oxidation Reaction Kinetics," *Journal of the American Chemical Society Au* 1,·no.·10 (2021): 1674-1687, <https://doi.org/10.1021/jacsau.1c00281>.

17. D. Kaminski, P. Poodt, E. Aret, et al., "Observation of a Liquid Phase with an Orthorhombic Orientational Order," *Physcial Review Letters* 96,·no.·5 (2006): 056102, <https://doi.org/10.1103/PhysRevLett.96.056102>.

18. K. G. Steenbergen, S. Lambie, N. Gaston, "Discerning Order from Chaos: Characterising the Surface Structure of Liquid Gallium," *Materials Horizons* 12,·no.·4 (2025): 1314-1322, <https://doi.org/10.1039/d4mh01415d>.

19. N. Lei, Z. Huang, S. A. Rice, "Surface Segregation and Layering in the Liquid–Vapor Interface of a Dilute Bismuth: Gallium Alloy," *The Journal of Chemical Physics* 104,·no.·12 (1996): 4802-4805, <https://doi.org/10.1063/1.471174>.

20. P. Hu, P. Hu, T. D. Vu, et al., "Vanadium Oxide: Phase Diagrams, Structures, Synthesis, and Applications," *Chemical Reviews* 123,·no.·8 (2023): 4353-4415, <https://doi.org/10.1021/acs.chemrev.2c00546>.

21. L. Artiglia, S. Agnoli, G. Granozzi, "Vanadium Oxide Nanostructures on Another Oxide: the Viewpoint from Model Catalysts Studies," *Coordination Chemistry Reviews* 301, (2015): 106-122, <https://doi.org/10.1016/j.ccr.2014.12.015>.

22. H. Yao, Y. Chen, Y. Wei, et al., "A Periodic DFT Study of Ammonia Adsorption on the V_2_O_5_ (001), V_2_O_5_ (010) and V_2_O_5_ (100) Surfaces: Lewis Versus Brönsted Acid Sites," *Surface Science* 606,·no.·21 (2012): 1739-1748, <https://doi.org/10.1016/j.susc.2012.07.020>.

23. Y. Liu, Q. Hu, D. Ma, et al., "Periodic DFT Study on the Adsorption and Deoxygenation Process of NH_3_ on V_2_O_5_ (001) Surface," *The Journal of The Minerals, Metals & Materials Society* 74,·no.·5 (2022): 1870-1877, <https://doi.org/10.1007/s11837-022-05256-6>.

24. F. Jiang, S. Wang, Y. Xu, et al., "Catalytic Activity for CO_2_ Hydrogenation Is Linearly Dependent on Generated Oxygen Vacancies over CeO_2_-Supported Pd Catalysts," *ChemCatChem* 14, (2022): e202200422, <https://doi.org/10.1002/cctc.202200422>.

25. W. Li, B. Liu, Q. Guo, et al., "Reaction-Induced Regioselective Reconstruction of Ni-Doped Ce(OH)_3_/CeO_2_ Enables Exceptional Activity and Selectivity for Reverse Water-Shift Reaction," *Nature Communications* 16,·no.·1 (2025): 7335, <https://doi.org/10.1038/s41467-025-62771-1>.

26. D. Ye, Z. Wu, T. Wang, et al., "Anti-Sintering Ni-W Catalytic Layer on Reductive Tungsten Carbides for Superior High-Temperature CO_2_ Reduction," *Advanced Materials* 37,·no.·29 (2025): e2504431, <https://doi.org/10.1002/adma.202504431>.

27. X. Zhou, C. Hansen, D. Isler, et al., "Tellurium-Induced Noble-Metal Reactivity in CO_2_ Hydrogenation Catalysts," *Journal of the American Chemical Society* 147,·no.·26 (2025): 22309-22313, <https://doi.org/10.1021/jacs.5c04185>.

28. Z. Ding, P. Li, Q. Chang, et al., "Ni^+^ Atoms Anchored In Situ on Ultrathin Ni-Phyllosilicate Nanosheet Ensure High-Efficient CO_2_ Reduction into CO at Moderate-Low Temperature," *Advanced Science*  (2025): e15872, <https://doi.org/10.1002/advs.202515872>.

29. Z. Zhu, K. Feng, C. Li, et al., "Stabilization of Exposed Metal Nanocrystals in High-Temperature Heterogeneous Catalysis," *Advanced Materials* 34,·no.·6 (2022): 2108727, <https://doi.org/10.1002/adma.202108727>.

30. F. Sun, C. Yan, Z. Wang, et al., "Ni/Ce-Zr-O Catalyst for High CO_2_ Conversion during Reverse Water Gas Shift Reaction (RWGS)," *International Journal of Hydrogen Energy* 40,·no.·46 (2015): 15985-15993, <https://doi.org/10.1016/j.ijhydene.2015.10.004>.

31. R. V. Gonçalves, L. L. R. Vono, R. Wojcieszak, et al., "Selective Hydrogenation of CO_2_ into CO on a Highly Dispersed Nickel Catalyst Obtained by Magnetron Sputtering Deposition: a Step towards Liquid Fuels," *Applied Catalysis B: Environmental* 209, (2017): 240-246, <https://doi.org/10.1016/j.apcatb.2017.02.081>.

32. R. Zhang, A. Wei, M. Zhu, et al., "Tuning Reverse Water Gas Shift and Methanation Reactions during CO_2_ Reduction on Ni Catalysts via Surface Modification by MoO_x_," *Journal of CO_2_ Utilization* 52, (2021): 101678, <https://doi.org/10.1016/j.jcou.2021.101678>.

33. B. Rutherford, C. Panaritis, E. Pahija, et al., "Ni Nanoparticles on Co_3_O_4_ Catalyze the Reverse Water Gas Shift with 95% CO Selectivity at 300°C," *Fuel* 348,·no.·384 (2023): 128523, <https://doi.org/10.1016/j.fuel.2023.128523>.

34. J. Guo, Z. Wang, J. Li, et al., "In-Ni Intermetallic Compounds Derived from Layered Double Hydroxides as Efficient Catalysts toward the Reverse Water Gas Shift Reaction," *ACS Catalysis* 12,·no.·7 (2022): 4026-4036, <https://doi.org/10.1021/acscatal.2c00671>.

35. Y. R. Dias, O. W. Perez-Lopez, "Carbon Dioxide Methanation over Ni-Cu/SiO_2_ Catalysts," *Energy Conversion and Management* 203, (2020): 112214, <https://doi.org/10.1016/j.enconman.2019.112214>.

36. D. Wang, Z. Yuan, X. Wu, et al., "Ni Single Atoms Confined in Nitrogen Doped Carbon Nanotubes for Active and Selective Hydrogenation of CO_2_ to CO," *ACS Catalysis* 13,·no.·10 (2023): 7132-7138, <https://doi.org/10.1021/acscatal.3c00767>.

37. H. Shen, Y. Dong, S. Yang, et al., "Identifying the Roles of Ce^3+^-OH and Ce-H in the Reverse Water-Gas Shift Reaction over Highly Active Ni-Doped CeO_2_ Catalyst," *Nano Research* 15,·no.·7 (2022): 5831-5841, <https://doi.org/10.1007/s12274-022-4207-8>.

38. N. Nityashreea, C. A. H. Priceb, L. Pastor-Perez, et al., "Garbon Stabilised Saponite Supported Transition Metal-Alloy Catalysts for Chemical CO_2_ Utilisation via Reverse Water Gas Shift Reaction," *Applied Catalysis B: Environmental* 261, (2020): 118214, <https://doi.org/10.1016/j.apcatb.2019.118241>.

39. S. Li, X. Liu, J. Ma, et al., "Develop High-Performance Cu-Based RWGS Catalysts by Controlling Oxide-Oxide Interface," *ACS Catalysis* 15,·no.·4 (2025): 3475-3486, <https://doi.org/10.1021/acscatal.4c07729>.

40. J. Lei, Z. Wu, D. Ye, et al., "Amorphous Silica Induced Loose CeO_2_ Clusters with Isolated Pt Atoms for Efficient Reverse‐Water Gas Shift Reaction," *Angewandte Chemie International Edition* 64,·no.·44 (2025): <https://doi.org/10.1002/anie.202511913>.
